# PINK1 is activated by mitochondrial membrane potential depolarization and stimulates Parkin E3 ligase activity by phosphorylating Serine 65

**DOI:** 10.1098/rsob.120080

**Published:** 2012-05

**Authors:** Chandana Kondapalli, Agne Kazlauskaite, Ning Zhang, Helen I. Woodroof, David G. Campbell, Robert Gourlay, Lynn Burchell, Helen Walden, Thomas J. Macartney, Maria Deak, Axel Knebel, Dario R. Alessi, Miratul M. K. Muqit

**Affiliations:** 1 MRC Protein Phosphorylation Unit, College of Life Sciences, University of Dundee, Dundee DD1 5EH, UK; 2 Division of Cell Signalling and Immunology, College of Life Sciences, University of Dundee, Dundee DD1 5EH, UK; 3 College of Medicine, Dentistry and Nursing, University of Dundee, Dundee DD1 5EH, UK; 4 Protein Structure and Function Laboratory, Cancer Research UK, London Research Institute, 44 Lincoln's Inn Fields, London WC2A 3LY, UK

**Keywords:** PINK1, Parkin, Parkinson's disease

## Abstract

Missense mutations in PTEN-induced kinase 1 (PINK1) cause autosomal-recessive inherited Parkinson's disease (PD). We have exploited our recent discovery that recombinant insect PINK1 is catalytically active to test whether PINK1 directly phosphorylates 15 proteins encoded by PD-associated genes as well as proteins reported to bind PINK1. We have discovered that insect PINK1 efficiently phosphorylates only one of these proteins, namely the E3 ligase Parkin. We have mapped the phosphorylation site to a highly conserved residue within the Ubl domain of Parkin at Ser^65^. We show that human PINK1 is specifically activated by mitochondrial membrane potential (Δψm) depolarization, enabling it to phosphorylate Parkin at Ser^65^. We further show that phosphorylation of Parkin at Ser^65^ leads to marked activation of its E3 ligase activity that is prevented by mutation of Ser^65^ or inactivation of PINK1. We provide evidence that once activated, PINK1 autophosphorylates at several residues, including Thr^257^, which is accompanied by an electrophoretic mobility band-shift. These results provide the first evidence that PINK1 is activated following Δψm depolarization and suggest that PINK1 directly phosphorylates and activates Parkin. Our findings indicate that monitoring phosphorylation of Parkin at Ser^65^ and/or PINK1 at Thr^257^ represent the first biomarkers for examining activity of the PINK1-Parkin signalling pathway *in vivo*. Our findings also suggest that small molecule activators of Parkin that mimic the effect of PINK1 phosphorylation may confer therapeutic benefit for PD.

## 2. Introduction

The human PINK1 gene encodes a 581 residue serine–threonine kinase unique among all protein kinases since it contains an N-terminal mitochondrial targeting motif (residues 1–34) [1,2]. The catalytic domain of PINK1 (residues 150–513) is not closely related to other protein kinases and is also unusual in that it possesses three unique insertions between the beta strands that make up the typical fold of the N-lobe of protein kinases [3]. PINK1 contains a conserved C-terminal non-catalytic region of unknown function (residues 514–581). Great excitement in understanding the regulation and function of this enzyme resulted from the 2004 landmark discovery that loss-of-function autosomal-recessive mutations in PINK1 caused early onset PD [2]. Subsequent studies in flies revealed that *Drosophila* PINK1 null mutants share many overlapping features with human PD, including motor deficits, neuronal loss and mitochondrial abnormalities [4,5]. Other work in *Drosophila* [6] suggests that PINK1 plays a role in regulating mitochondrial dynamics, for example over-expression of PINK1 enhances mitochondrial fission whilst loss of PINK1 leads to excess fusion.

Recent work in mammalian cells provides further links between PINK1 and the mitochondria. Current data suggest that following recruitment of PINK1 to the mitochondrial membrane via its N-terminal targeting sequence, it is subsequently proteolysed between residues Ala^103^–Phe^104^ by the mitochondrial rhomboid protease, PARL [7–10], resulting in a processed form of PINK1 which is rapidly degraded by the 20S proteasome [1,11]. In response to mitochondrial membrane potential (Δψm) depolarization, for example induced by the uncoupling agent carbonyl cyanide *m*-chlorophenyl hydrazone (CCCP), a marked stabilization of full length PINK1 at the mitochondria is observed [12–15]. How CCCP stabilizes full-length PINK1 is not known, but one proposal is that Δψm depolarization leads to relocalization of PINK1 from the inner to the outer mitochondrial membrane where it is no longer accessible by PARL [8].

Despite considerable research, we still have limited knowledge of the mechanism by which PINK1 kinase activity is regulated, what substrates it might phosphorylate physiologically and how this links to PD. In our hands and those of many other groups recombinant PINK1 expressed in mammalian cells is inactive, which has limited the ability to use traditional biochemical approaches to identify PINK1 substrates. Here, we have exploited our recent discovery that insect orthologues of PINK1, including *Tribolium castaneum* PINK1 (TcPINK1), are catalytically active when expressed in *Escherichia coli* [3] to investigate whether TcPINK1 was capable of phosphorylating 11 proteins encoded by genes linked to Mendelian-inherited PD as well as seven proteins reported to bind PINK1. This excitingly revealed that PINK1 had a marked ability to phosphorylate one of these proteins, namely the RING E3 ligase, Parkin.

Autosomal-recessive inherited mutations in Parkin are one of the most frequent causes of familial PD, especially young-onset forms [16]. Since previous genetic analysis in *Drosophila* [4,5] and mammalian cells [17] had suggested significant links between PINK1 and Parkin and human patients with mutations in either of these enzymes display very similar clinical symptoms [18], we decided to further investigate the phosphorylation of Parkin by PINK1. Our findings suggest that both insect as well as human PINK1 directly phosphorylate a highly conserved serine residue (Ser^65^) lying within the N-terminal Ubiquitin-like (Ubl) domain. We also present evidence that CCCP and other agonists that depolarize the Δψm specifically activate human PINK1 enabling it to phosphorylate Parkin at Ser^65^ *in vivo*. We further establish the functional importance of Ser^65^ phosphorylation by demonstrating that phosphorylation of Ser^65^ leads to activation of Parkin E3 ligase activity. We also present data that indicate that once PINK1 is activated following CCCP treatment, it autophosphorylates at several residues, including Thr^257^, and this is associated with an electrophoretic mobility band-shift on an 8 per cent sodium dodecyl sulphate (SDS)-polyacrylamide gel. Our results indicate that PINK1 is activated following CCCP treatment and that Parkin is a PINK1 substrate. Our findings suggest that monitoring phosphorylation of Parkin at Ser^65^ and/or PINK1 at Thr^257^ would be useful as a reporter for PINK1-Parkin pathway activity. Our findings also suggest that small molecule activators of Parkin may confer a novel therapeutic approach for PD.

## 3. Results

### 3.1. Insect PINK1 phosphorylates Parkin *in vitro*

**Figure 1.**
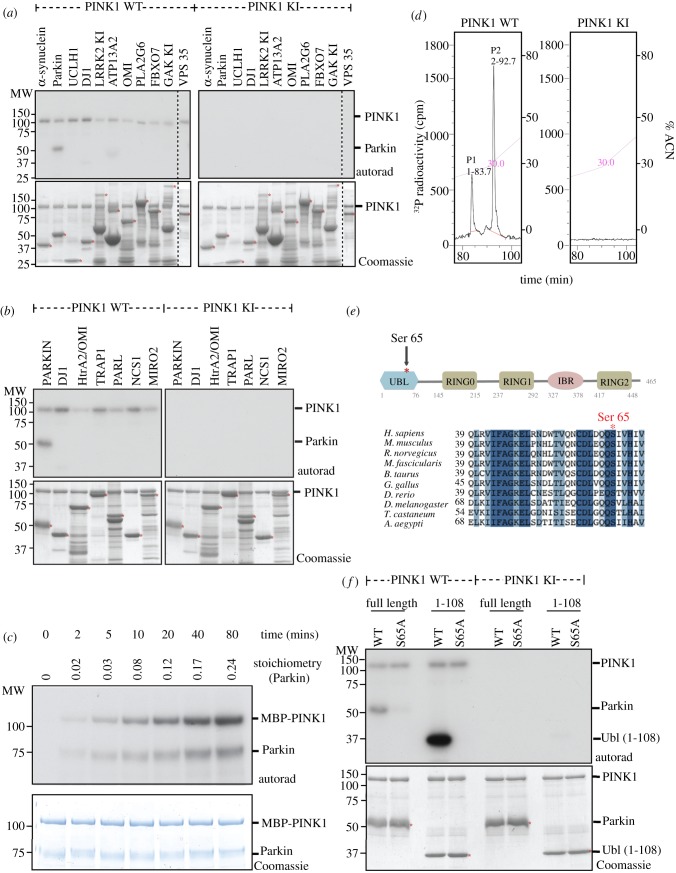
(*Overleaf*.) TcPINK1 phosphorylates human Parkin at Ser^65^ *in vitro*. (*a*) The indicated PD-linked proteins (1 μM) were incubated with either full-length MBP-fusion of wild-type TcPINK1 (1–570) or kinase-inactive (KI) TcPINK1 (D359A) (0.5 μg) and [γ-^32^P] ATP for 30 min. Assays were terminated by addition of SDS loading buffer and separated by SDS-PAGE. Proteins were detected by Colloidal Coomassie blue staining (lower panel) and incorporation of [γ-^32^P] ATP was detected by autoradiography (upper panel). Similar results were obtained in three independent experiments. Fine dividing lines indicate that reactions were resolved on separate gels. The substrate bands on the Coomassie gel are denoted with a small red asterisk. All substrates were of human sequence and expressed in *E. coli* unless otherwise indicated. Tags on the substrates used for this experiment were glutathione s-transferase (GST)-α-synuclein, Parkin (no tag as His-SUMO tag cleaved off), His-UCHL1, GST-DJ1, GST-LRRK2 KI (1326-end D2017A), MBP-ATP13A2, GST-Omi, MBP-PLA2G6, GST-FBX07, GST-GAK-kinase-inactive (D191A), VPS35 (no tag as GST-tag cleaved off). (*b*) As in (*a*) except that proteins reported to interact with PINK1 were tested as PINK1 substrates. Human DJ1, Omi, TRAP1, PARL, NCS1 and Miro2 were expressed in *E. coli* with an N-terminal GST tag. Similar results were obtained in three independent experiments. (*c*) Timecourse of phosphorylation of Parkin by wild-type TcPINK1. MBP-TcPINK1 (0.5 μg) was incubated in the presence of GST-Parkin (1 μg) and [γ-^32^P] ATP for the times indicated and assays terminated by addition of SDS loading buffer. Samples were subjected to SDS-PAGE and proteins detected by Colloidal Coomassie blue staining (lower panel) and incorporation of [γ-^32^P] ATP was detected by autoradiography (upper panel). Gel pieces were quantified by Cerenkov counting for calculation of the stoichiometry of Parkin phosphorylation. Similar results were obtained in two independent experiments. (*d*) Mapping of phosphopeptides on Parkin after phosphorylation by TcPINK1 *in vitro*. Full-length GST-Parkin (1 μg) was incubated with 2 μg of either wild-type TcPINK1 (1–570) or KI TcPINK1 (D359A) in the presence of Mg^2+^[γ-^32^P] ATP for 60 min. Assays were terminated by addition of LDS loading buffer and separated by SDS-PAGE. Proteins were detected by Colloidal Coomassie blue staining and phosphorylated Parkin was digested with trypsin. The resultant peptides were separated by reverse phase HPLC on a Vydac C_18_ column (Vydac 218TP5215) equilibrated in 0.1% (v/v) trifluoroacetic acid and the column developed with an acetonitrile gradient (diagonal line). The flow rate was 0.2 ml min^−1^ and fractions (0.1 ml each) were collected and analysed for ^32^P radioactivity by Cerenkov counting. Two major ^32^P-labelled peaks (P1, P2) were identified following incubation with wild-type TcPINK1 (left). No peaks were identified following incubation with kinase-inactive TcPINK1 (right). (*e*) Schematic of domain organization of Parkin illustrating that Ser^65^ lies within the Ubl domain (upper panel) and sequence alignment of residues around Ser^65^ in human Parkin and a variety of lower organisms showing high degree of conservation. Abbreviations: Ubl, ubiquitin-like; IBR, in-between-RING; RING, really interesting new gene. (*f*) Mutation of Ser^65^Ala (S65A) abolishes Parkin phosphorylation by TcPINK1. Full-length wild-type TcPINK1 (1–570) and kinase inactive TcPINK1 (D359A) against wild-type or S65A mutants of full-length Parkin, or the isolated Ubl-domain-containing N-terminal fragment (residues 1–108). The indicated substrates (2 μM) were incubated in the presence of the indicated enzyme (1 μg) and [γ-^32^P] ATP for 30 min. Assays were terminated by addition of SDS loading buffer and separated by SDS-PAGE. Proteins were detected by Colloidal Coomassie blue staining (lower panel) and incorporation of [γ-^32^P] ATP was detected by autoradiography (upper panel).

As some of the known PD-linked proteins may function in a signalling network [19], we tested whether catalytically active recombinant insect TcPINK1 could directly phosphorylate 11 different PD-linked proteins and seven putative PINK1 interacting proteins (figure 1*a*,*b*). Strikingly, this revealed that wild-type but not kinase-inactive TcPINK1 phosphorylated full-length Parkin, but not any of the other proteins tested, including Omi [20], TRAP1 [21] or Miro2 [22,23] (figure 1*a*,*b*).

### 3.2. Insect PINK1 phosphorylates Parkin at Ser^65^, a highly conserved residue within the Ubl domain

TcPINK1 phosphorylated Parkin in a time-dependent manner reaching a maximal stoichiometry of phosphorylation of approximately 0.25 moles of ^32^P-phosphate per mole of protein (figure 1*c*). ^32^P-labelled Parkin was digested with trypsin and analysed by chromatography on a C_18_ column. Two major ^32^P-labelled phosphopeptides were observed (figure 1*d*). A combination of solid-phase Edman sequencing and mass spectrometry revealed that both of these encompassed variants of a peptide phosphorylated at Ser^65^ (see electronic supplementary material, figure S1A and B). Ser^65^ is located within the N-terminal Ubl domain of Parkin and is highly conserved from mammals to invertebrates (figure 1*e*). Mutating Ser^65^ to Ala prevented phosphorylation of full-length Parkin or an N-terminal Parkin fragment containing the isolated Ubl domain (residues 1–108) by TcPINK1 thereby confirming that this residue represents the major site of PINK1 phosphorylation (figure 1*f*). The isolated Ubl domain of Parkin (residues 1–108) was phosphorylated to a significantly higher stoichiometry than full-length Parkin in a parallel experiment (figure 1*f*). We also attempted to assess phosphorylation of a longer C-terminal-truncated fragment of Parkin (residues 1–383) but were unable to do this rigorously as this fragment was unstable (data not shown).

### 3.3. PINK1 phosphorylation of Parkin at Ser^65^ mediates activation of Parkin E3 ligase activity

**Figure 2.**
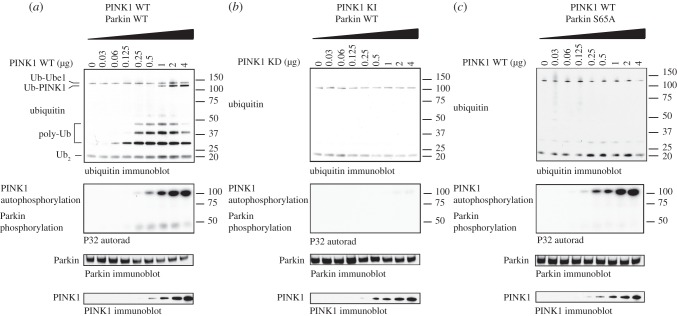
PINK1 phosphorylation of Parkin at Ser^65^ mediates activation of Parkin E3 ligase activity. Wild-type (*a*) but not kinase-inactive (*b*) PINK1 activates wild-type Parkin, but does not affect the activity of Ser^65^Ala (S65A) mutant Parkin (*c*). Two micrograms of wild-type or S65A Parkin were incubated with indicated amounts of wild-type or kinase-inactive (D359A) MBP-TcPINK in a kinase reaction (50 mM Tris-HCl (pH 7.5), 0.1 mM ethylene glycol tetraacetic acid (EGTA), 10 mM MgCl_2_, 1% 2-mercaptoethanol and 0.1 mM [γ-^32^P] ATP (approx. 500 cpm pmol^−1^) (in parallel to confirm the phosphorylation) for 60 min. The ubiquitylation reaction was then initiated by addition of ubiquitylation assay components (50 mM Tris-HCl (pH 7.5), 0.05 mM EGTA, 10 mM MgCl_2_, 0.5% 2-mercaptoethanol, 0.12 μM human recombinant E1 purified from Sf21 insect cell line, 1 μM human recombinant UbcH7 purified from *E. coli*, 0.05 mM Flag-Ubiquitin (MW approx. 9.5 kDa) (Boston Biochem) and 2 mM ATP). Reactions were terminated after 60 min by addition of SDS-PAGE loading buffer and resolved by SDS-PAGE. Ubiquitin, Parkin and PINK1 were detected using anti-FLAG, anti-Parkin and anti-MBP antibodies, respectively. Incorporation of [γ-^32^P] ATP was detected by autoradiography (lower panel). Ubiquitin attached to the E1 (Ub-Ube1) and ubiquitin dimer (Ub_2_) formation occurred in the assay in all conditions (*a*–*c*). Ubiquitylation of PINK1 (Ub-PINK1) is indicated (*a*). Formation of polyubiquitin chains (poly-Ub) upon Parkin activation (*a*) is indicated. As mentioned in §4, further work is required to establish the nature of these chains and whether they are linked to UbcH7. Representative of five independent experiments.

A recent study provided strong evidence that the Ubl domain of Parkin acts as an auto-inhibitory domain by binding to a region within the C-terminus thereby suppressing catalytic activity [24]. Given that Ser^65^ lies within the core of the Ubl domain, we hypothesized that phosphorylation of Ser^65^ might relieve the autoinhibition thereby activating the E3 ligase activity of Parkin. To investigate this, we set up an E3 ligase auto-ubiquitylation assay to assess Parkin catalytic activity using an approach that has been described previously employing highly purified full length recombinant Parkin expressed in *E. coli* with no epitope tags that can interfere with the autoinhibitory effect of the Ubl domain [24]. Prior to undertaking the E3 ligase activity assay, we phosphorylated Parkin with increasing levels of TcPINK1 in the presence of ^32^P-adenosine triphosphate (ATP) so that we could verify PINK1 was phosphorylating Parkin (middle panels in figure 2). To assess Parkin E3 ligase activity, aliquots of these reactions were added to a reaction containing E1 ubiquitin-activating ligase, UbcH7 conjugating E2 ligase, ubiquitin and Mg-ATP. After 60 min the reactions were terminated with SDS sample buffer in the presence of dithiothreitol (DTT) and reactions analysed by immunoblot analysis with antibodies that detect ubiquitin, Parkin and TcPINK1. In the absence of PINK1 phosphorylation we confirmed previous findings and found that Parkin displayed no significant E3 ligase activity and no evidence of formation of polyubiquitin chains were observed (lane 1 on figure 2*a*). Excitingly, when increasing levels of TcPINK1 were added to the reaction at concentrations in which phosphorylation of Parkin was detected (middle panel of figure 2*a*), we observed the marked dose-dependent appearance of non-DTT-reducible low molecular weight polyubiquitylated species migrating between approximately 30 and 50 kDa (top panel of figure 2*a*). Consistent with this being mediated by phosphorylation of Parkin at Ser^65^ by PINK1, the appearance of polyubiquitin chains was inhibited by introducing a point mutation in PINK1 that ablates catalytic activity (figure 2*b*) or by mutating Ser^65^ in Parkin to a non-phosphorylatable Ala residue (figure 2*c*).

### 3.4. Evidence that human PINK1 phosphorylates Parkin at Ser^65^ *in vivo*

**Figure 3.**
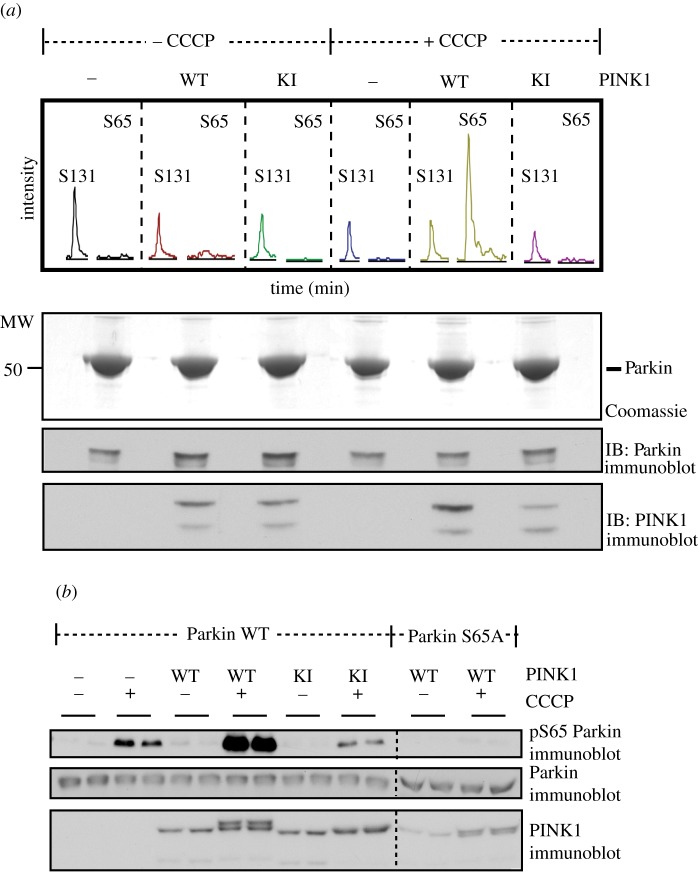
Human Parkin Ser^65^ is a substrate of human PINK1 upon CCCP stimulation. (*a*) Confirmation by mass spectrometry that Ser^65^ of human Parkin is phosphorylated by CCCP-induced activation of human wild-type PINK1-FLAG. Flp-In T-Rex HEK293 cells expressing FLAG-empty, wild-type PINK1-FLAG, and kinase-inactive PINK1-FLAG (D384A) were co-transfected with HA-Parkin, induced with doxycycline and stimulated with 10 μM of CCCP for 3 h. Whole-cell extracts were obtained following lysis with 1% Triton and approximately 30 mg of whole-cell extract were subjected to immunoprecipitation with anti-HA-agarose and run on 10% SDS-PAGE and stained with colloidal Coomassie blue. Coomassie-stained bands migrating with the expected molecular mass of HA-Parkin were excised from the gel, digested with trypsin, and subjected to high performance liquid chromatography with tandem mass spectrometry (LC-MS-MS) on an LTQ-Orbitrap mass spectrometer. Extracted ion chromatogram analysis of Ser^131^ and Ser^65^ phosphopeptide (3^+^ R.NDWTVQNCDLDQQ**S**IVHIVQRPWR.K+P). The total signal intensity of the phosphopeptide is plotted on the *y*-axis and retention time is plotted on the *x*-axis. The *m*/*z* value corresponding to the Ser^131^ phosphopeptide was detected in all conditions whilst that of the Ser^65^ phosphopeptide was only detected in samples from wild-type PINK1-FLAG-expressing cells following CCCP treatment. (*b*) Characterization of Parkin phospho-Ser^65^ antibody. Flp-In T-Rex HEK293 cells expressing FLAG-empty, wild-type PINK1-FLAG, and kinase-inactive PINK1-FLAG were co-transfected with untagged wild-type (WT) or Ser^65^Ala (S65A) mutant Parkin, induced with doxycycline and stimulated with 10 μM of CCCP for 3 h. 0.25 mg of 1% Triton whole-cell lysate were subjected to immunoprecipitation with anti-Parkin antibody (S966C) covalently coupled to protein G Sepharose and then immunoblotted with anti-phospho-Ser^65^ antibody in the presence of dephosphorylated peptide. Ten per cent of the immunoprecipitate (IP) was immunoblotted with total anti-Parkin antibody. Twenty five micrograms of whole cell lysate was immunoblotted with total PINK1 antibody.

To investigate whether human PINK1 has the potential to phosphorylate Parkin, we over-expressed full-length human Parkin in human HEK293 Flp-In TRex cells stably expressing wild-type human PINK1, or kinase-inactive human PINK1 (D384A) (figure 3*a*). Cells were treated with or without the mitochondrial uncoupling agent, CCCP, for 3 h—conditions that induce stabilization and activation of PINK1 at the mitochondria (see §2 and also figure 6). Parkin was immunoprecipitated and phosphorylation site analysis undertaken by mass spectrometry. This strikingly revealed that Parkin was phosphorylated at Ser^65^, but only in cells expressing wild-type human PINK1 that had been stimulated with CCCP (figure 3*a*). No detectable phosphorylation of Ser^65^ was observed in the absence of CCCP treatment or in cells expressing kinase-inactive PINK1 (figure 3*a*). This result suggests that CCCP treatment might activate PINK1 enabling it to phosphorylate Parkin (this is explored below). We also detected phosphorylation of a previously reported phosphorylation site (Ser^131^). In contrast to Ser^65^, phosphorylation of Ser^131^ was constitutive and not modulated by CCCP or PINK1 (figure 3*a*). We failed to detect phosphorylation of Parkin at another previously reported site (Thr^175^) [25].

We next raised a phospho-specific antibody that specifically recognized Parkin phosphorylated at Ser^65^ and used this to confirm that Parkin phosphorylation at Ser^65^ is markedly induced by CCCP (3 h treatment) in HEK293 cells expressing human wild-type but not kinase-inactive PINK1 *in vivo* (figure 3*b*). Moreover, mutation of Ser^65^ to Ala abolished recognition of Parkin in CCCP-treated cells overexpressing wild-type PINK1 confirming the specificity of the antibody we have generated. Interestingly, using the phosphospecific Ser^65^ antibody that is much more sensitive than the mass spectrometry approach employed in analysis of data in figure 3*a*, we also observed phosphorylation of Parkin Ser^65^ in cells not over-expressing PINK1 treated with CCCP suggesting endogenous PINK1 present in HEK293 is also able to phosphorylate Parkin (figure 3*b*).

**Figure 4.**
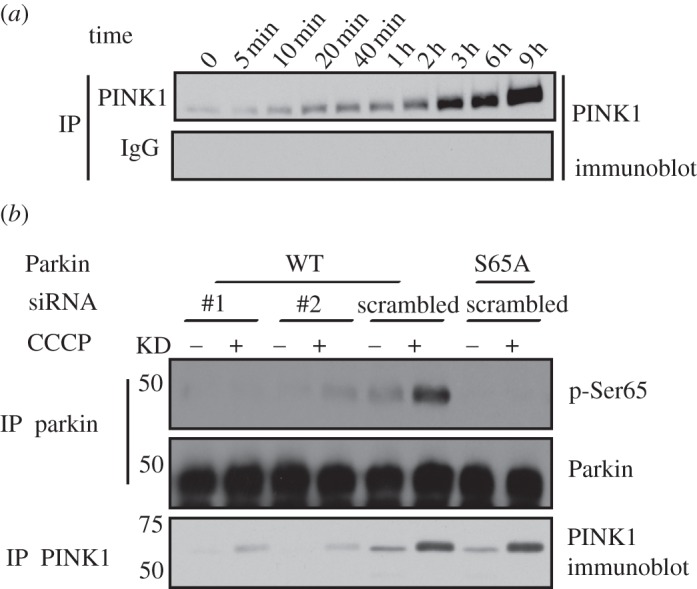
Knock-down of endogenous PINK1 abrogates Parkin Ser^65^ phosphorylation. (*a*) Timecourse of endogenous PINK1 stabilization by CCCP treatment. HEK293 cells were stimulated at the indicated time points with 10 μM of CCCP. One milligram of whole-cell lysates were immunoprecipitated with anti-PINK1 antibody (S085D) or pre-immune IgG covalently coupled to protein G Sepharose and resolved by 8% SDS-PAGE. Immunoblotting was performed with total PINK1 antibody (Novus). Representative of three independent experiments. (*b*) Knock-down of endogenous PINK1 abrogates Parkin Ser^65^ phosphorylation. HEK293 cells were co-transfected with PINK1 siRNA (#1 or #2) or scrambled siRNA (scrambled) and untagged wild-type (WT) or Ser^65^Ala (S65A) mutant Parkin as indicated using TransFectin reagent (Bio-Rad). Forty-eight hours post-transfection, cells were treated with or without 10 μM CCCP for 3 h. 0.25 mg of 1% Triton whole-cell lysate were subjected to immunoprecipitation with GST-Parkin antibody (S966C) covalently coupled to protein G Sepharose and then immunoblotted with anti-phospho-Ser^65^ antibody in the presence of dephosphorylated peptide. Five per cent of the IP was immunoblotted with total anti-Parkin antibody. 0.25 mg of whole-cell lysates were immunoprecipitated with anti-PINK1 antibody (S085D) and immunoblotted with anti-PINK1 antibody (Novus). Representative of three independent experiments.

To test whether we could detect endogenous PINK1 we subjected HEK293 cell lysates to immunoprecipitation employing a PINK1 antibody that we raised against a recombinant fragment of human PINK1 (residues 175–250) and immunoblotted the immunoprecipitates with a commercial PINK1 antibody (raised against residues 175–250; Novus Biologicals). This revealed the presence of a band that migrated at the size predicted for endogenous PINK1 and that was strikingly stabilized by CCCP in a time-dependent manner (up to 9 h; upper band figure 4*a*). This protein was not observed in control immunoprecipitates undertaken with pre-immune Immunoglobulin G (IgG; lower band figure 4*a*). Consistent with this band representing endogenous PINK1 it was significantly reduced when cells were transfected for 48 h with two independent PINK1 siRNA oligos but not scrambled siRNA (figure 4*b*). Importantly, siRNA-mediated knockdown using two different siRNA probes targeting PINK1 severely abrogated phosphorylation of Parkin Ser^65^, suggesting that the endogenous PINK1 protein does indeed regulate Parkin phosphorylation at Ser^65^ (figure 4*b*).

### 3.5. Human PINK1 isolated from CCCP-treated cells is capable of phosphorylating Parkin *in vitro*

**Figure 5.**
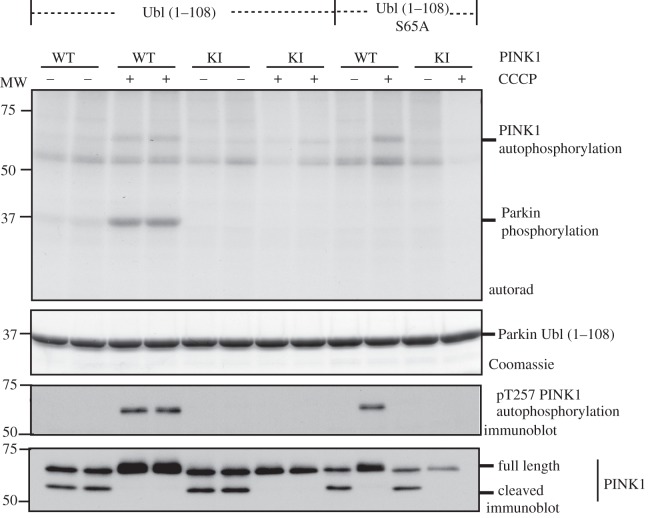
Human PINK1 directly phosphorylates Parkin Ser^65^ upon CCCP stimulation *in vitro*. Flp-In T-Rex HEK293 cells expressing wild-type PINK1-FLAG, and kinase-inactive PINK1-FLAG were induced to express protein by addition of 0.1 μg ml^−1^ of doxycycline in the culture medium for 24 h. Cells were then treated with 10 μM of CCCP for 3 h and lysates subjected to sub-cellular fractionation. Five milligrams of mitochondrial lysate was subjected to immunoprecipitation with anti-FLAG agarose and used in an *in vitro* radioactive kinase assay with [γ-^32^P]-Mg^2+^ATP and *E. coli* expressed recombinant GST-Parkin Ubl domain (residues 1–108) (Ubl) and mutant GST-Parkin (residues 1–108) Ser^65^Ala (Ubl S65A), purified from *E. coli*. One half of the assay reaction was run on a 10% SDS-PAGE and was subjected to autoradiography. Colloidal Coomassie stained gel shows equal loading of recombinant substrate. The other half of the reaction was immunoblotted with anti-phospho-Thr^257^ PINK1 and total PINK1 antibodies following 8% SDS-PAGE.

Our results suggest that human PINK1 is activated by CCCP treatment and thereby rendered capable of phosphorylating Parkin at Ser^65^. To test this, we first immunoprecipitated wild-type or kinase-inactive PINK1 from the mitochondrial fraction of cells treated with CCCP and tested to see whether it could phosphorylate the Ubl domain of Parkin *in vitro* (figure 5). This revealed that wild-type PINK1 isolated from CCCP-treated cells but not from non-treated cells could indeed phosphorylate the Ubl domain of Parkin (figure 5). Importantly, kinase-inactive PINK1 isolated from CCCP-stimulated cells failed to phosphorylate the Ubl domain of Parkin. Mutation of Ser^65^ to Ala also prevented wild-type PINK1 isolated from CCCP-stimulated cells from phosphorylating the Ubl domain of Parkin (figure 5). These observations indicate that CCCP treatment is indeed leading to the activation of human PINK1 enabling it to directly phosphorylate Parkin at Ser^65^.

### 3.6. Further evidence that CCCP promotes activation of human PINK1 enabling it to autophosphorylate at Thr^257^ and other residues

**Figure 6.**
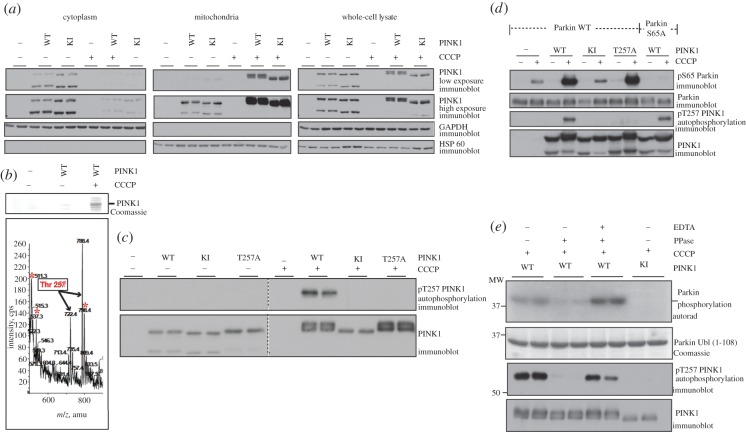
Identification and characterization of a novel autophosphorylation site of PINK1 induced by the mitochondrial uncoupling agent CCCP. (*a*) CCCP induces a band-shift in wild-type but not kinase-inactive PINK1. Flp-In T-Rex HEK293 cell lines stably expressing FLAG alone, wild-type or kinase-inactive PINK1-FLAG were induced to express protein by addition of 0.1 μg ml^−1^ of doxycycline in the culture medium for 24 h. Cells were then treated with 10 μM of CCCP for 3 h and lysates subjected to sub-cellular fractionation. Twenty-five micrograms of cytoplasmic or mitochondrial lysate were resolved by 8% SDS-PAGE. Relative purity of the fractions was confirmed using cytoplasmic and mitochondrial markers, namely GAPDH and HSP60, respectively. Whole-cell extracts from the same cells were also made in parallel using 1% Triton lysis as described in the methods. In mitochondrial and whole-cell extracts, both wild-type and kinase-inactive PINK1 became stabilized by CCCP but a band-shift was noted for wild-type PINK1 which was revealed to be a doublet on lower exposure. The upper band was absent from kinase-inactive PINK1 treated with CCCP. (*b*) Identification of Thr^257^ phosphorylation site on PINK1. Flp-In T-Rex HEK293 cell lines stably expressing FLAG alone, or wild-type PINK1-FLAG were treated with DMSO or 10 μM of CCCP for 3 h. Recombinant PINK1 was immunoprecipitated from 10 mg of mitochondrial extract for each condition using anti-FLAG-agarose and subjected to 4–12% gradient SDS-PAGE and stained with colloidal Coomassie blue. The Coomassie-stained bands migrating with the expected molecular mass of PINK1-FLAG were excised from the gel, digested with trypsin, and subjected to precursor-ion scanning mass spectroscopy. The major phosphopeptide that is indicated ‘Thr^257^’ was seen from cells expressing wild-type PINK1-FLAG treated with CCCP and this was not seen in bands from the other two conditions. The figure shows the signal intensity (cps, counts of ions per second) of the HPO_3_^−^ ion (−79 Da) seen in negative precursor ion scanning mode versus the ion distribution (*m*/*z*) for the Thr^257^ phosphopeptide. The observed values of 722.4 and 788.4 are for the VALAGEYGAVTYR and VALAGEYGAVTYRK variants, respectively, of the Thr^257^ peptide as [M-2H]^2−^ ions. Other phosphopeptides marked with an asterisk were observed but we were unable to assign phosphorylation site(s). (*c*) Evidence that CCCP induces PINK1 auto-phosphorylation using a phospho-specific Thr^257^ antibody. 0.5 mg of mitochondrial extracts (treated with DMSO or 10 μM of CCCP for 3 h) of Flp-In T-Rex stable cell lines expressing FLAG empty, wild-type PINK1-FLAG, kinase-inactive PINK1-FLAG (D384A) and phospho-mutant Thr^257^Ala (T257A) were immunoprecipitated with anti-FLAG agarose and resolved by 8% SDS-PAGE. Blots were probed with anti-phospho-Thr^257^ PINK1 antibody and anti-PINK1 antibody. (*d*) Mutation of Thr^257^Ala PINK1 does not affect Parkin Ser^65^ phosphorylation. Flp-In T-Rex HEK293 cells expressing FLAG-empty, wild-type PINK1-FLAG, kinase-inactive PINK1-FLAG and T257A PINK1-FLAG were co-transfected with untagged wild-type (WT) or Ser^65^Ala (S65A) mutant Parkin, induced with doxycycline and stimulated with 10 μM of CCCP for 3 h. 0.25 mg of 1% Triton whole-cell lysate were subjected to immunoprecipitation with anti-Parkin antibody (S966C) covalently coupled to protein G Sepharose and then immunoblotted with anti-phospho-Ser^65^ Parkin antibody in the presence of dephosphorylated peptide. Ten per cent of the IP was immune-blotted with total anti-Parkin antibody. One milligram of 1% Triton whole-cell lysate were immunoprecipitated with anti-FLAG agarose and resolved by 8% SDS-PAGE. Blots were probed with anti-phospho-Thr^257^ PINK1 and anti-PINK1 antibody. (*e*) PINK1 dephosphorylation by lambda phosphatase inhibits PINK1 activity. C-terminal-FLAG tagged wild-type or kinase-inactive (D384A) PINK1 were immunoprecipitated from 5 mg of mitochondrial enriched extracts using anti-FLAG agarose beads. Wild-type PINK1 was incubated with or without 1000 U of lambda phosphatase or treated with lambda phosphatase along with 50 mM EDTA. Kinase-inactive PINK1 was incubated in buffer alone without lambda phosphatase. The beads were washed thrice in 50 mM Tris pH 7.5, 0.1 mM EGTA and then used in an *in vitro* kinase assay with GST-Parkin Ubl (1–108) as the substrate. Samples were analysed as described in legend to figure 1.

We next studied the cellular localization and electrophoretic mobility of wild-type and kinase-inactive human PINK1 in response to CCCP (see §5). Similar to previous observations [12–15] in non-CCCP-treated cells, full-length as well as a truncated form of wild-type and kinase-inactive PINK1 was present in both the cytoplasmic and mitochondrial fractions (figure 6*a*). N-terminal Edman sequencing of the truncated form of PINK1 confirmed that it commenced at residue 104 (see electronic supplementary material, figure S2) consistent with previous work indicating that human PINK1 is proteolysed between residues Ala^103^–Phe^104^ by the mitochondrial rhomboid protease, PARL [7–10]. A 3 h CCCP treatment induced a marked increase in the levels of the full-length form of PINK1 associated with the mitochondria, which was accompanied by a large reduction in cytoplasmic levels of PINK1 (figure 6*a*). We also observed that CCCP treatment led to a significant increase in levels of full-length PINK1 in whole-cell extracts (figure 6*a*) consistent with CCCP stabilizing full-length PINK1. Levels of full-length kinase-inactive PINK1 were also stabilized following CCCP treatment (figure 6*a*).

We also noticed that concomitant to inducing activation of PINK1 and thereby Parkin phosphorylation (figures 3–5), CCCP treatment induced a significant decrease in the electrophoretic mobility (‘band-shift’) of the wild-type but not kinase-inactive PINK1 (figure 6*a*). Many protein kinases become capable of autophosphorylation at many residues when activated and this frequently results in a band-shift of the wild type but not a catalytically inactive mutant that is incapable of autophosphorylation. This therefore prompted us to investigate whether CCCP stimulated phosphorylation of any residues on PINK1. We undertook mass spectrometric phosphopeptide analysis of wild-type and kinase-inactive full-length PINK1 after immunoprecipitation from mitochondrial fractions of CCCP-treated cells. This revealed that several residues of PINK1 were phosphorylated in CCCP-treated cells at low stoichiometry making the identification of phosphorylation sites challenging. Thus far we have only been able to unambiguously identify one of these phosphorylation sites, that corresponds to Thr^257^ (figure 6*b* and electronic supplementary material, figure S3). Employing a phosphospecific Thr^257^ antibody that we raised, we were able to confirm that CCCP treatment markedly stimulated phosphorylation of wild-type but not kinase-inactive PINK1 at Thr^257^ (figure 6*c*), suggesting that this residue is an autophosphorylation site. Mutation of Thr^257^ to Ala abolished detection of phosphorylated PINK1 confirming the specificity of the Thr^257^ antibody (figure 6*c*). We also confirmed that the active PINK1 we isolated from CCCP-treated cells and used to phosphorylate the Ubl domain of Parkin (figure 5) was indeed phosphorylated at Thr^257^ (panel 3 in figure 5). Parkin Ser^65^ phosphorylation was still observed in CCCP-treated cells expressing the PINK1 Thr^257^Ala mutant (figure 6*d*) suggesting that phosphorylation of this residue is not required for CCCP-induced PINK1 activation *in vivo*. Moreover, the Thr^257^Ala mutation did not prevent the CCCP-induced band-shift indicating that phosphorylation of this residue is not critical for this (figure 6*c*,*d*). Thr^257^ is located within the second insert region (residues 247–270) and, like many autophosphorylation sites in other protein kinases, is not highly conserved between species. Nevertheless monitoring phosphorylation of this residue could serve as a useful marker for PINK1 activity at least until better reporters for PINK1 activation are identified.

We next investigated how phosphatase treatment affects CCCP-induced PINK1 activity. We found that lambda phosphatase treatment of PINK1 isolated from CCCP-treated cells induced complete dephosphorylation of Thr^257^, and also resulted in a significant inhibition of PINK1 activity as judged by its ability to phosphorylate Parkin. Addition of the lambda phosphatase inhibitor EDTA prevented dephosphorylation of Thr^257^ and loss of the ability of PINK1 to phosphorylate Parkin. Given that Thr^257^ phosphorylation is dispensable for Parkin Ser^65^ phosphorylation *in vivo* (figure 6*d*), this suggests that phosphorylation of PINK1 at additional sites other than Thr^257^ may be important in mediating the activation of PINK1 induced by CCCP (figure 6*e*). We also observed that phosphatase treatment did not collapse the CCCP-induced band-shift (figure 6*e*), indicating that either phosphatase-resistant sites or another type of protein modification mediates the band-shift.

### 3.7. Timecourse of PINK1 activation, autophosphorylation and phosphorylation of Parkin

**Figure 7.**
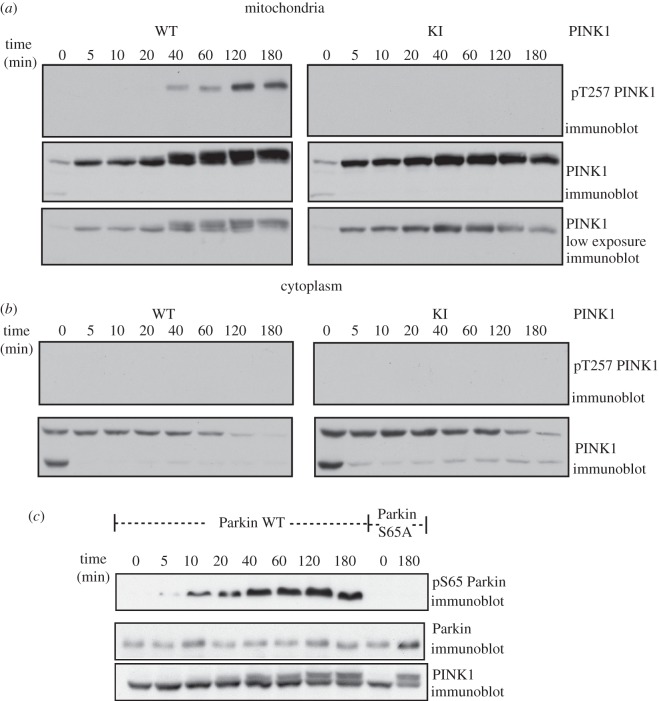
Timecourse of CCCP-induced activation of PINK1. (*a*) Timecourse of PINK1 autophosphorylation *in vivo*. Flp-In T-Rex HEK293 cells stably expressing PINK1-FLAG wild-type and kinase-inactive (D384A) were stimulated at the indicated time points with 10 μM of CCCP. 0.5 mg of mitochondrial extracts were immunoprecipitated with anti-FLAG agarose and resolved by 8% SDS-PAGE. Immunoblotting was performed with anti-phospho-Thr^275^ antibody or total PINK1 antibody. (*b*) No time-dependent activation of cytoplasmic PINK1 *in vivo*. As in (*a*) cytoplasmic extracts were obtained at the indicated time-points and immunoprecipitated with anti-FLAG agarose and resolved by 8% SDS-PAGE. Immunoblotting was performed with PINK1 anti-phospho-Thr^275^ antibody or total PINK1 antibody. (*c*) Timecourse of Parkin Ser^65^ phosphorylation *in vivo*. Flp-In T-Rex HEK293 cells stably expressing wild-type PINK1-FLAG were co-transfected with untagged wild-type (WT) or Ser^65^Ala (S65A) mutant Parkin; induced with doxycycline and stimulated with 10 μM of CCCP at the indicated time points. 0.25 mg of 1% Triton whole-cell lysate were subjected to immunoprecipitation with anti-Parkin antibody (S966C) covalently coupled to protein G Sepharose and then immunoblotted with anti-phospho-Ser^65^ antibody in the presence of dephosphorylated peptide. One per cent of the IP was immunoblotted with total anti-Parkin antibody. 1.5 mg of whole-cell extracts were immunoprecipitated with anti-FLAG agarose and resolved by 8% SDS-PAGE. Immunoblotting was performed with total PINK1 antibody.

We next investigated the timecourse of the PINK1 stabilization, band-shift, autophosphorylation of Thr^257^ and ability of PINK1 to phosphorylate Parkin following CCCP treatment. This revealed that the stabilization of full-length PINK1 at the mitochondria is rapid with significant stabilization seen within 5 min of CCCP treatment and is maximal by 40 min and then sustained for up to 3 h (figure 7*a*). Loss of the cleaved form of PINK1 observed in the cytosol is particularly rapid and almost disappears within 5 min of CCCP treatment (figure 7*b*). However, the appearance of the band-shift and autophosphorylation of Thr^257^ occurred more slowly and was observed only after 40 min of CCCP treatment and was sustained for up to 3 h (figure 7*a*). There was no phosphorylation of Thr^257^ or band-shift of cytoplasmic-associated PINK1 indicating that mitochondrial association is required for this (figure 7*b*). In contrast, monitoring Parkin Ser^65^ phosphorylation using the phospho-specific antibody against phospho-Ser^65^ indicated that Parkin Ser^65^ phosphorylation occurs at 5 min (figure 7*c*) and becomes maximal and sustained from 40 min onwards. This suggests that the kinetics of PINK1 activation against its substrate are significantly faster than the kinetics of PINK1 autophosphorylation.

### 3.8. PINK1 is activated specifically by depolarization of the inner mitochondrial membrane potential

**Figure 8.**
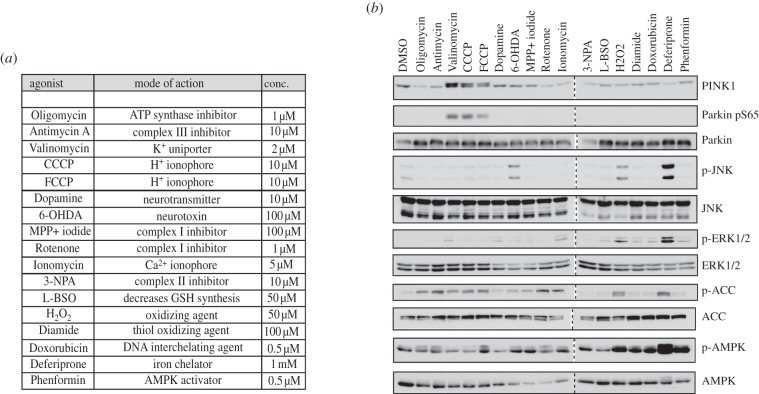
PINK1 is specifically activated by inducers of mitochondrial membrane depolarization. (*a*) Table of agonists tested. (*b*) PINK1 phosphorylation of Parkin at Ser^65^ is specific to mitochondrial uncouplers. Flp-In T-Rex HEK293 cells expressing wild-type PINK1-FLAG were co-transfected with untagged wild-type Parkin, induced with 0.1 μg ml^−1^ doxycycline for 24 h and stimulated with the indicated agonists for 3 h except for Deferiprone (24 h treatment). 0.25 mg of 1% Triton whole-cell lysate was subjected to immunoprecipitation with anti-Parkin antibody (S966C) covalently coupled to protein G Sepharose and then immunoblotted with anti-phospho-Ser^65^ antibody in the presence of dephosphorylated peptide. Ten per cent of the IP was immunoblotted with total anti-Parkin antibody. Twenty-five micrograms of whole-cell lysate was immunoblotted with total PINK1 antibody (Novus), phospho-JNK (CST), total-JNK (CST), phospho-ERK1/2, total-ERK1/2 (CST), phospho-ACC (CST), total ACC (CST), phospho-AMPK (CST) and total AMPK (CST). Representative of two independent experiments.

Within the inner mitochondrial membrane (IMM), the electron transport chain transfers electrons through a series of oxidation–reduction reactions coupled to the transfer of protons across the IMM and this efflux creates a proton electrochemical gradient known as the protomotive force. The protomotive force drives the re-entry of protons through the proton channel of the F_1_F_0_-ATP synthase crucial for ATP production and comprises mainly of an electrical component—the mitochondrial membrane potential (Δψm)—and a transmembrane pH gradient [26]. CCCP dissipates both Δψm and the pH gradient leading to impaired mitochondrial ATP synthesis. To determine the mechanism of activation of PINK1, we tested a panel of agonists that have previously been reported to disrupt mitochondria by diverse modes of action (figure 8*a*). Under the conditions tested only the proton ionophores CCCP, FCCP and the potassium uniporter valinomycin were able to induce activation of PINK1 leading to Parkin Ser^65^ phosphorylation (figure 8*b*). In contrast to CCCP and FCCP, valinomycin depolarizes the Δψm but does not affect the pH gradient suggesting that PINK1 is specifically activated by loss of the Δψm. We did not observe any effect of an inhibitor of ATP synthase (oligomycin) or various inhibitors of the electron transport chain complexes that have previously been implicated in neurodegeneration models (MPP+, rotenone, 3-nitropropionic acid).

## 4. Discussion

Our data indicate that PINK1 is activated following mitochondrial membrane potential (Δψm) depolarization and that PINK1 directly phosphorylates Parkin at Serine 65 (Ser^65^) inducing its activation. Previous observations indicating that *Drosophila* dPINK1 and dParkin null flies have similar degenerative phenotypes [4,5] and that over-expression of Parkin rescues the phenotype of dPINK1 null *Drosophila* (but not the converse) are consistent with PINK1 acting upstream of Parkin [4,5]. The ability of PINK1 to regulate mitochondrial dynamics in mammalian cells as well as *Drosophila* has also been suggested to be dependent upon Parkin [6,17,27–29]. The finding that humans with loss-of-function mutations in either PINK1 or Parkin display indistinguishable clinical presentation of PD also argues in favour of a major connection between PINK1 and Parkin in humans [18].

Parkin belongs to the RING-in-between-RING (RBR) family of ubiquitin ligases and recent work suggests that Parkin as well as other RBR family members may function as RING/HECT hybrids that use the HECT-preferred E2, UbcH7, and catalyse transfer of ubiquitin to substrates via an intermediate thioester-linked ubiquitin adduct on a conserved cysteine in the RING2 domain (Cys^431^) [30]. It was recently proposed that the Ubl domain of Parkin acts as an auto-inhibitory domain by binding to the C-terminal region and preventing catalytic activity by sterically preventing ubiquitin binding to the C-terminus [24]. However, the mechanism of activation of Parkin remained unknown [24].

Inspection of all the available structures, both nuclear magnetic resonance (NMR) and crystal, reveals that Ser^65^ may sometimes lie within the fifth β-strand that makes up the ubiquitin-like fold and is partially exposed to the surface (PDB code 2KNB (solution, mouse) [31]; PDB code 1IYF (solution, human) [32]; see electronic supplementary material, figure S4) or sometimes may lie within a loop adjacent to the strand (PDB code 1MG8 (solution, mouse) [33]; PDB code 2ZEQ (crystal, mouse) [34]) suggesting some conformational flexibility around this element. It is therefore possible that upon interaction with PINK1, the Ubl domain undergoes a local conformational change enabling PINK1 to gain access to Ser^65^ for phosphorylation. One possibility is that phosphorylation at Ser^65^ may perturb the β-strand formation in the Ubl domain leading to loss of the autoinhibition and subsequent activation of Parkin. Indeed previous work has shown that this region of the Ubl domain in which Ser^65^ lies is critical for maintaining the autoinhibition [24]. In future work it would be critical to study the biophysical interaction between PINK1 and Parkin, define the interacting domains in more detail and establish how phosphorylation of Ser^65^ impacts on this interaction leading to the activation of Parkin. The finding that phosphorylation of full-length Parkin is much less efficient than phosphorylation of the isolated Ubl-domain suggests that other factors might regulate the ability of PINK1 to phosphorylate Parkin at Ser^65^, which would be interesting to investigate in future studies.

In this study, we have assessed Parkin E3 ligase activity employing an auto-ubiquitylation assay that establishes that following Ser^65^ phosphorylation, Parkin in the presence of an E1, and the E2, UbcH7, is capable of inducing the formation of short polyubiquitylated chains. In future work, it would be vital to repeat these assays employing physiological Parkin substrate(s) to confirm that Ser^65^ phosphorylation of Parkin does indeed enhance the ubiquitylation of a genuine Parkin substrate. In our assay, it is possible that these short polyubiquitylated chains are covalently attached to the E2, UbcH7, since in a recent study of the RBR family member, HHARI, similar ubiquitin species were produced by HHARI catalytic activity, which were attributed to HHARI E3-dependent ubiquitylation of UbcH7 [30]. Further work will be required to determine the nature of these ubiquitin species and whether or not they are linked to UbcH7. It will also be exciting to test the effect of disease mutants located within and out with the Ubl domain as well as a mutant of cysteine (Cys^431^) on the catalytic activity of Parkin.

**Figure 9.**
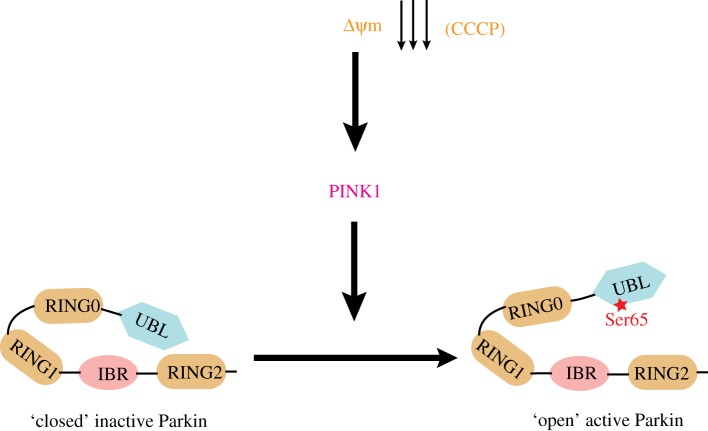
Model of Parkin activation by PINK1. Under basal conditions Parkin is kept in a closed inactive conformation by Ubl-mediated autoinhibition. Following mitochondrial depolarization, PINK1 phosphorylates Parkin at Ser^65^ thereby relieving Parkin autoinhibition and enabling Parkin to become active to ubiquitylate target substrates.

Our data provide fundamental insights into the regulation of Parkin and elaborate a signalling pathway that may be central to neuronal loss in PD (figure 9). Our data would suggest that loss-of-function mutations in PINK1 would lead to suppression of Parkin E3 ligase activity and result in reduced ubiquitylation of Parkin's targets. This may explain why over-expression of Parkin in dPINK1 null *Drosophila* restores ubiquitylation of targets and rescues the null phenotype [4,5]. It is possible that the key Parkin targets are located at the mitochondria and indeed several candidate mitochondrial substrates for Parkin have been proposed, including Mitofusin1 [35], VDAC1 [12] and more recently PARIS [36] and Miro [37]. In future work, it would be vital to test whether phosphorylation of Parkin at Ser^65^ influences its ability to ubiquitylate these or other targets and define how this links to PD. Our data also suggest that small molecules that bind to and disrupt the Ubl domain-C-terminus auto-inhibitory interface may activate Parkin in a similar manner to Ser^65^ phosphorylation. If Parkin inactivation occurs in sporadic PD patients in addition to those harbouring PINK1 mutations, then such small molecule activators could hold significant therapeutic promise in combating disease progression in PD.

In previous work, employing a positional scanning peptide library approach, we elaborated an artificial peptide substrate termed PINKtide that had the sequence WIpYRR**S**PRRR. This was phosphorylated by an insect orthologue, TcPINK1, albeit weakly with a *V*_max_ of 8 U mg^−1^ and a *K*_m_ of 493 μM [3]. This contrasts with optimal peptides for other active protein kinases that can usually be phosphorylated with a *V*_max_ of 100–1000 U mg^−1^ and *K*_m_ of less than 10 μM. Mutation of the +1 Pro in PINKtide to other residues tested inhibited phosphorylation, suggesting that this residue might represent a key determinant for PINK1 phosphorylation [3]. However, the sequence encompassing Ser^65^ of Parkin, DLDQQ**S**IVHI, is quite dissimilar from PINKtide and does not possess a +1 Pro residue. Our Parkin data suggest that a +1 Pro residue is not an essential determinant for PINK1 phosphorylation. Previous studies, based on co-immunoprecipitation and co-localization experiments [38,39], have reported that PINK1 and Parkin bind to each other and therefore it is possible that additional docking interactions between PINK1 and Parkin enable Ser^65^ to be efficiently phosphorylated. This may also explain why a short peptide, encompassing Ser^65^, synthesized by our laboratory was not significantly phosphorylated by TcPINK1 (data not shown).

There has been one previous report that human PINK1 isolated from non-CCCP unstimulated cells can directly phosphorylate Parkin at a single threonine residue, Thr^175^, [25]. In that study a deletion fragment of PINK1 spanning residues 200–581 was used that would be predicted to be missing approximately the first 50 amino acids of the N-lobe of the PINK1 kinase domain including the conserved glycine-rich loop motif (residues 163–169), which in other kinases is essential for coordinating ATP. This construct of PINK1 would therefore not be expected to be active in our opinion. Moreover, in that report, the kinase-inactive mutant of the PINK1 [200–581] fragment still exhibited substantial kinase activity towards Parkin [25]. Taken together these findings indicate that the phosphorylation of Thr^175^ observed in this study was likely to be mediated by a contaminating kinase. Our experiments have also identified Ser^131^ as a phosphorylation site in Parkin that is constitutively phosphorylated and not influenced by PINK1 or CCCP (figure 3*a*). Ser^131^ lies within the linker region of Parkin between the Ubl and RING0 domain and unlike Ser^65^ is not fully conserved in lower organisms (e.g. leucine in *Drosophila*). A previous *in vitro* study has suggested that Ser^131^ may be phosphorylated by Cdk5 [40], and further work would be required to define the importance of this phosphorylation site.

Our findings suggest that the full-length form of PINK1 becomes rapidly stabilized within 5 min of CCCP treatment and this also coincides with the disappearance of the cleaved form of PINK1 (figure 7*a*). PINK1 also becomes activated at 5 min and reaches maximal activity at 40 min as assessed by monitoring phosphorylation of Parkin Ser^65^ within cells (figure 7*c*). However, the timecourse of PINK1 autophosphorylation at Thr^257^ takes longer, requiring around 40 min with activation then being sustained for at least up to 3 h (figure 7*a*). It is not uncommon for kinases to exhibit differential kinetics of catalytic activity for autophosphorylation as compared with substrate phosphorylation. The kinetics for kinase activity against a substrate generally occurs faster than autophosphorylation and is regarded as a more reliable read-out of kinase activation. Interestingly, previous work has suggested that Parkin can translocate to the mitochondria following CCCP treatment and that this is enhanced when PINK1 is co-expressed in cells [12–15]. The kinetics of Parkin translocation when PINK1 is co-expressed is 5 min compared with 30 min when PINK1 is absent [14]. Given this is similar to the kinetics of Parkin phosphorylation, it would be interesting to test whether phosphorylation of Ser^65^ influences the kinetics of Parkin translocation to mitochondria in cells.

What drives the stabilization of full length PINK1 at the mitochondria and destabilization of cleaved PINK1 is unknown at present. One proposal is that the PARL protease-mediated cleavage is rapidly inhibited following CCCP-induced depolarization in mitochondria thereby leading to stabilization of the full-length form of PINK1 [9], while another report suggests that in the presence of CCCP, PINK1 is relocated to the outer mitochondrial membrane rendering it inaccessible by PARL [8]. The striking disappearance of the cleaved form of PINK1 in the cytoplasm within 5 min (figure 7*b*) also suggests that CCCP could trigger rapid degradation of this form of PINK1 or else inhibit maturation and processing of PINK1 in the cytosol by PARL-independent proteolysis. In future work, it would be important not only to investigate how full-length PINK1 is stabilized and the cleaved form of PINK1 destabilized, but also to determine whether simple recruitment of PINK1 to mitochondria is sufficient to induce its activation or whether additional depolarization and/or stabilization of the full-length form of PINK1 is a pre-requisite for subsequent activation of PINK1.

It would also be essential to discover the mechanism by which PINK1 is activated at the mitochondria following CCCP treatment. Our data suggest that activation of PINK1 can be observed after immunoprecipitation and extensive washing of the immunoprecipitate, which may be consistent with a covalent modification. Lambda phosphatase treatment substantially reduced PINK1 activation suggesting that phosphorylation is required for full activation (figure 6*e*). In future work, it would be essential to establish what are the key phosphorylation and/or other covalent modifications induced by CCCP and whether these are responsible for triggering activation of PINK1. Given the specificity of activation of PINK1 by agonists that depolarize the Δψm (figure 8*a*,*b*), it is also possible that PINK1 may become associated with a non-covalent activator at the mitochondrial membrane, such as another protein or a small molecule second messenger that is recruited following Δψm depolarization. It would also be interesting to investigate whether CCCP-mediated generation of an intermediate molecular species, such as reactive oxygen species, could modify PINK1 leading to its activation.

Our data suggest that PINK1, like many kinases, autophosphorylate at Thr^257^ and probably other residues we have not identified after it is activated. Based on multiple sequence alignments, Thr^257^ appears to lie within the centre of a region, which is predicted to be an insertion loop and so would likely be accessible for phosphorylation [3]. This is also associated with an electrophoretic mobility shift on a polyacrylamide gel after CCCP treatment of wild-type but not kinase-inactive PINK1, which would be incapable of autophosphorylation (figure 6*a*,*b*). It should be noted that this band-shift was best observed by resolving proteins on an 8 per cent isocratic polyacrylamide gel and was much less pronounced on gradient gels (see electronic supplementary material, figure S3). This may explain why the band-shift of wild-type PINK1 following CCCP treatment has not been reported before. Although our data suggest that autophosphorylation of Thr^257^ is not critical for triggering the activation of PINK1 we still feel that phospho-antibodies that recognize Thr^257^ are likely to be a useful reporter of human PINK1 activation, at least until a biomarker can be generated against the genuine trigger of PINK1 activation, which is currently unknown.

Given the large body of evidence implicating mitochondrial dysfunction in PD [26], it would be important to explore in subsequent work whether Ser^65^ and perhaps Thr^257^ phosphorylation could have utility as specific biomarkers for PD progression. It would also be interesting to look at Ser^65^ and Thr^257^ phosphorylation in transgenic mouse models of PD (e.g. α-synuclein) to determine whether this pathway is implicated in other genetic forms of PD. It would also be important to examine Ser^65^ and Thr^257^ phosphorylation in brains and cell lines of patients with Parkin and PINK1 mutations and, more importantly, sporadic PD. In future work, it would be important to raise state-of-the-art biomarker-grade monoclonal antibodies against these residues.

In conclusion, we have added important new information that supports the notion that PINK1 and Parkin function in a common signalling pathway. Our data suggest that Δψm depolarization induces stabilization and activation of PINK1 at the mitochondria enabling it to directly phosphorylate Parkin at Ser^65^ within the N-terminal Ubl domain leading to activation of the Parkin E3 ligase activity. We also provide evidence that, once activated, PINK1 autophosphorylates at several residues and this is associated with an electrophoretic band-shift on a polyacrylamide gel. We have identified one of these autophosphorylated residues as Thr^257^ and provided evidence that this could serve as a reporter for PINK1 activation. Our findings provide reagents and a framework for exciting follow-up studies. Firstly, it will be crucial to understand how CCCP and other agents that induce Δψm depolarization activate PINK1. It will also be essential to identify the key substrates that Parkin ubiquitylates in response to Ser^65^ phosphorylation. Hopefully such information could provide valuable clues as to how disruption of the PINK1-Parkin signalling pathway leads to PD and whether this pathway is also disrupted in patients with the sporadic form of the disease. These studies are imperative as they could lead to new ideas for therapies to treat PD in the future, for example by developing a chemical probe that activates Parkin by disrupting the Ubl-domain-mediated autoinhibition of Parkin.

## 5. Material and methods

### 5.1. Reagents and general methods

Tissue culture reagents were from Life Technologies. [γ-^32^P] ATP was from Perkin Elmer. The Flp-In T-Rex HEK293 cell line was from Invitrogen and stable cell lines were generated according to the manufacturer's instructions by selection with hygromycin. Restriction enzyme digests, DNA ligations and other recombinant DNA procedures were performed using standard protocols. All mutageneses were carried out using the QuikChange site-directed mutagenesis method (Stratagene) with KOD polymerase (Novagen). All DNA constructs were verified by DNA sequencing, which was performed by The Sequencing Service, School of Life Sciences, University of Dundee, using DYEnamic ET terminator chemistry (Amersham Biosciences) on Applied Biosystems automated DNA sequencers. Parkin cDNA was cloned by PCR from a human brain cDNA library and verified by sequencing (Ser223Pro polymorphic variant; GenBank: BAA25751.1). DNA for mammalian cell transfection were amplified in *E. coli* DH5α strain and plasmid preparation was done using Qiagen Maxi prep Kit according to manufacturers protocol. DNA for bacterial protein expression were transformed into *E. coli* BL21 DE3 RIL (codon plus) cells (Stratagene).

### 5.2. Cell culture and stimulations

Flp-In T-Rex stable cell lines were cultured using DMEM (Dulbecco's modified Eagle's medium) supplemented with 10 per cent FBS (foetal bovine serum), 2 mM l-Glutamine, 1X Pen/Strep, 15 μg ml^−1^ of Blasticidin and 100 μg ml^−1^ of Hygromycin. Cell transfections of HA tagged Parkin or untagged Parkin were performed using the polyethyleneimine method [41]. Cultures were induced to express protein by addition of 0.1 μg ml^−1^ of Doxycycline in the medium for 24 h. To uncouple mitochondria, cells were treated with 10 μM CCCP (Sigma) dissolved in DMSO for the indicated times. An equivalent volume of DMSO was used as a control. In addition, cells were incubated with the following agonists for 3 h including: 1 μM Oligomycin (Sigma), 10 μM Antimycin A (Sigma), 2 μM Valinomycin (Sigma), 10 μM FCCP (Sigma), 10 μM Dopamine (Sigma), 100 μM 6-Hydroxy-dopamine (Sigma), 100 μM MPP+ (Sigma), 1 μM rotenone (Sigma), 5 μM ionomycin (Sigma), 10 μM 3-nitropropionic acid (Sigma), 50 μM l-BSO (Sigma), 50 μM hydrogen peroxide (Sigma), 100 μM Diamide (Sigma), 0.5 μM Doxorubicin (Sigma), 0.5 μM Phenformin (Sigma). Cells were treated with 1 mM Deferiprone (3-Hydroxy-1,2-dimethyl-4 (1H)-pyridone; Sigma) for 24 h.

### 5.3. siRNA knock-down of PINK1 expression

To knock down PINK1 gene expression, HEK293 cells were transfected separately with two sets of Mission siRNA oligos (Sigma) designated as siRNA #1 (5′-CCAUCAAGAUGAUGUGGAATT-3′) or siRNA #2 (5′-CAGAGAAGUGUUGUGUGGATT-3′) and scrambled control siRNA (Sigma). Cells were transfected using TransFectin Lipid Reagent (Bio-Rad) and incubated for 48 h before CCCP treatment. The final concentration of siRNA was 30 nM. For detection of phosphorylated pSer^65^-Parkin, 10 μg of untagged wild-type or Ser^65^Ala Parkin was co-transfected with siRNAs, respectively, into the cells according to the above method.

### 5.4. Buffers and methods for mammalian cell lysis

Cells were lysed and fractionated by the indicated buffer and methods: whole-cell lysis using buffer: 50 mM Tris/HCl (pH 7.4), 1 mM EGTA, 1 mM EDTA, 1% (w/v) 1 mM sodium orthovanadate, 10 mM sodium β-glycerophosphate, 50 mM NaF, 5 mM sodium pyrophosphate, 0.27 M sucrose, 1 mM benzamidine and 2 mM phenylmethylsulfonyl fluoride (PMSF) and 1% (v/v) Triton X-100. Lysates were clarified by centrifugation at 13 000 r.p.m. for 10 min at 4°C and the supernatant was collected. Mitochondrial fractionation: cells were lysed in buffer containing 250 mM sucrose, 20 mM HEPES, 3 mM EDTA, 1% (w/v) 1 mM sodium orthovanadate, 10 mM sodium β-glycerophosphate, 50 mM NaF, 5 mM sodium pyrophosphate, pH 7.5 and protease inhibitor cocktail (Roche) at 4°C. Cells were disrupted using a glass hand-held homogenizer (20 passes) and the lysate was clarified by centrifuging for 10 min at 800*g* at 4°C. The supernatant was further centrifuged at 16 600*g* for 10 min. The resultant supernatant served as the cytosolic fraction. The pellet containing the mitochondrial fraction was resuspended in buffer containing 1 per cent Triton X-100 and centrifuged at 13 000 r.p.m. for 10 min. This supernatant contained solubilized mitochondrial proteins. All lysates were snap-frozen at −80°C until use. Protein concentration was determined using the Bradford method (Thermo Scientific) with BSA as the standard.

### 5.5. Buffers for *E. coli* protein purification

Lysis buffer contained 50 mM Tris-HCl (pH 7.5), 150 mM NaCl, 1 mM EDTA, 1 mM EGTA, 5% (v/v) glycerol, 1% (v/v) Triton X-100, 0.1% (v/v) 2-mercaptoethanol, 1 mM benzamidine and 0.1 mM PMSF. Wash buffer contained 50 mM Tris-HCl (pH 7.5), 500 mM NaCl, 0.1 mM EGTA, 5% (v/v) glycerol, 0.03% (v/v) Brij-35, 0.1% (v/v) 2-mercaptoethanol, 1 mM benzamidine and 0.1 mM PMSF. Equilibration buffer contained 50 mM Tris-HCl (pH 7.5), 150 mM NaCl, 0.1 mM EGTA, 5% (v/v) glycerol, 0.03% (v/v) Brij-35, 0.1% (v/v) 2-mercaptoethanol, 1 mM benzamidine and 0.1 mM PMSF. Elution buffer was equilibration buffer with the addition of 12 mM maltose. Storage buffer was equilibration buffer with the addition of 0.27 M sucrose and glycerol—PMSF and benzamidine were omitted.

### 5.6. Protein purification from *E. coli*

Full length wild-type and kinase-inactive TcPINK1 was expressed in *E. coli* as maltose binding protein (MBP) fusion protein and purified as described previously [3]. Briefly, BL21 Codon+ transformed cells were grown at 37°C to an OD_600_ of 0.3, then shifted to 16°C and induced with 250 μM IPTG (isopropyl β-d-thiogalactoside) at OD_600_ of 0.5. Cells were induced with 250 μM IPTG at OD 0.6 and were further grown at 16°C for 16 h. Cells were pelleted at 4000 r.p.m., and then lysed by sonication in lysis buffer. Lysates were clarified by centrifugation at 30 000*g* for 30 min at 4°C followed by incubation with 1 ml per litre of culture of amylose resin for 1.5 h at 4°C. The resin was washed thoroughly in wash buffer, then equilibration buffer, and proteins were then eluted. Proteins were dialysed overnight at 4°C into storage buffer, snap-frozen and stored at −80°C until use.

MBP-ATP13A2 and MBP-PLA2G6 were purified by similar methods. GST-α-synuclein, GST-Parkin, GST-DJ1, GST-LRRK2 kinase inactive (KI) (1326-end D2017A), GST-Omi, GST-GAK KI (D191A), GST-FBX07, untagged VPS35 (GST cleaved), GST-TRAP1, GST-PARL, GST-NCS1 and GST-Miro2 were purified by similar methods except that recombinant GST-fusion proteins were affinity purified on glutathione-Sepharose and eluted with buffer containing 20 mM glutathione. GST-VPS35 was cleaved with GST-PreScission protease at 4°C overnight. His-UCHL1 was obtained from Ubiquigent (UK). His-SENP1 catalytic domain (residues 415–643) was purified as follows: transformed BL21 cells were grown in LB (Luria Broth), 50 µg ml^−1^ carbenicillin until OD_600_ = 0.6, then induced with 300 µM IPTG (isopropyl β-d-1-thiogalactopyranoside) and expressed overnight at 15°C. The cells were collected, lysed and the protein was purified using Ni^2+^-nitriloacetic acid-Sepharose chromatography, followed by dialysis into 50 mM HEPES pH 7.5, 10% glycerol, 150 mM NaCl, 1 mM DTT).

Untagged Parkin (His-SUMO cleaved) was expressed and purified using a modified protocol from Helen Walden's laboratory [24]. BL21 cells were transformed with His-SUMO-tagged Parkin constructs, overnight cultures were prepared and used to inoculate 12 × 1 l LB medium, 50 µg ml^−1^ carbenicillin, 0.25 mM ZnCl_2_. The cells were grown at 37°C until the OD_600_ was 0.4 and the temperature was dropped to 16°C. At OD_600_ = 0.8 expression was induced with 25 µM IPTG. After overnight incubation the cells were collected and lysed in 75 mM Tris pH 7.5, 500 mM NaCl, 0.2 per cent Triton X-100, 25 mM imidazole, 0.5 mM tris(2-carboxyethyl)phosphine (TCEP), 1 mM Pefablok, 10 µg ml^−1^ Leupeptin. After sonication and removal of insoluble material, His-SUMO-Parkin was purified via Ni^2+^-NTA-Sepharose chromatography. The protein was collected by elution with 400 mM imidazole in 50 mM Tris, pH 8.2, 200 mM NaCl, 10 per cent glycerol, 0.03 per cent Brij 35, 0.5 mM TCEP. This was dialysed twice against 50 mM Tris pH 8.2, 200 mM NaCl, 10 per cent glycerol, 0.5 mM TCEP in the presence of His-SENP1 415–643 at a ratio of 1 mg His-SENP1 per 5 mg His-SUMO-Parkin. The protease, the His-SUMO tag and any uncleaved protein was removed by two subsequent incubations with Ni^2+^–NTA–Sepharose. The cleaved Parkin was further purified in 50 mM Tris, pH 8.2, 200 mM NaCl, 20 per cent glycerol, 0.03 per cent Brij, 0.5 mM TCEP over a Superdex 200 column.

### 5.7. Antibodies

The antibody against PINK1 phospho-Thr^257^ (S114D) was generated by injection of the KLH (keyhole-limpet haemocyanin)-conjugated phospho-peptide CAGEYGAVpTYRKSKR (where pT is phospho-threonine) into sheep and was affinity-purified by positive and negative selection against the phospho- and de-phospho-peptides, respectively. The antibody against Parkin phospho-Ser^65^ (S210D) was generated by injection of the KLH-conjugated phospho-peptide RDLDQQpSIVHIVQR (where pS is phospho-serine) into sheep and was affinity-purified by positive and negative selection against the phospho- and de-phospho-peptides, respectively. The antibody against total Parkin (S966C) was raised in sheep against the recombinant GST-Parkin full-length protein and successively affinity-purified by positive and negative selection against recombinant fusion protein and GST, respectively. The antibody against total PINK1 (S085D) was raised in sheep against a recombinant GST-PINK1 fragment (residues 175–250) and successively purified by positive and negative selection against recombinant fusion protein and GST, respectively. Anti-human PINK1 rabbit polyclonal (residues 175–250) antibody was obtained from Novus Biologicals; anti-GAPDH mouse monoclonal from Millipore; anti-Parkin mouse monoclonal (Santa Cruz). Anti-HSP60 rabbit polyclonal, Anti-phospho-JNK, Anti-total-JNK, Anti-phospho-ERK1/2, Anti-total-ERK1/2, Anti-phospho-ACC, Anti-total ACC, Anti-phospho-AMPK and Anti-total AMPK antibodies were all obtained from Cell Signaling Technology. Anti-FLAG agarose beads were obtained from Sigma.

### 5.8. Immunoprecipitation and immunoblotting

Immunoprecipitation of recombinant PINK1-FLAG was undertaken by standard methods with anti-FLAG agarose beads (Sigma); of HA-Parkin with anti-HA agarose beads (Sigma); of untagged Parkin with anti-Parkin antibody (S966C) covalently conjugated to protein G-Sepharose; and of endogenous PINK1 with anti-PINK1 antibody (S085D) or pre-immune IgG (negative control) covalently conjugated to protein G-Sepharose. Immunoprecipitates, as well as cell lysates in SDS sample buffer were subjected to SDS-PAGE and transferred to PVDF membranes. For immunoblotting, membranes were incubated for 60 min with 1 per cent Tris-buffered saline with Tween (TBST) containing either 5% (w/v) skimmed milk powder (for total antibodies) or 5% (w/v) BSA (for phospho-specific antibodies). The antibodies were then incubated in the same buffer overnight at 4°C with the indicated primary antibodies. Sheep total and phospho-specific antibodies were used at a concentration of 1 μg ml^−1^, whereas commercial antibodies were diluted 1000-fold. The incubation with phospho-specific sheep antibodies was performed with the addition of 10 μg ml^−1^ of the dephosphopeptide antigen used to raise the antibody. Blots were washed with 0.1 per cent TBST and incubated with secondary HRP-conjugated antibodies in 5 per cent skimmed milk for 60 min. After repeated washes, the signal was detected with enhanced chemiluminescence and the X-ray films were processed in a Konica Minolta Medical SRX-101 film processor.

### 5.9. Kinase assays

In assays using *E. coli*-expressed wild-type or kinase-inactive (D359A) MBP-TcPINK1, reactions were set up in a volume of 40 μl, with substrates at 1 μM and kinase at 0.5 μg in 50 mM Tris-HCl (pH 7.5), 0.1 mM EGTA, 10 mM MgCl_2_, 2 mM DTT and 0.1 mM [γ-^32^P] ATP (approx. 500 cpm pmol^−1^). Assays were incubated at 30°C with shaking at 1200 r.p.m. and terminated after the indicated time by addition of SDS sample buffer. In mammalian HEK293 immunoprecipitation kinase assays, C-terminal-FLAG tagged wild-type or KI (D384A) PINK1 was immunoprecipitated from 5 mg of mitochondrial enriched extracts using anti-FLAG agarose beads and activity measured in a reaction volume of 40 μl consisting of 50 mM Tris-HCl (pH 7.5), 0.1 mM EGTA, 10 mM MgCl_2_, 2 mM DTT, 0.1 mM [γ-^32^P] ATP (2000 cpm pmol^−1^) and 5 μM of indicated substrate. Assays were incubated at 30°C with shaking at 1200 r.p.m. and terminated after 30 min by addition of SDS sample buffer. For all assays, reaction mixtures were resolved by SDS-PAGE. Proteins were detected by Coomassie staining and gels were imaged using an Epson scanner and dried completely using a gel dryer (Bio-Rad). Incorporation of [γ-32P] ATP into substrates was analysed by autoradiography using Amersham Hyper-Film.

### 5.10. Lambda phosphatase assay

C-terminal-FLAG tagged wild-type or kinase-inactive (D384A) PINK1 were immunoprecipitated from 5 mg of mitochondrial enriched extracts using anti-FLAG agarose beads. Wild-type PINK1 was incubated with or without 1000 U of lambda phosphatase (New England Biolabs) in a reaction volume of 40 μl consisting of 50 mM Tris pH 7.5, 1 mM MnCl_2_ and 2 mM DTT. In addition, wild-type PINK1 was treated with 1000 U of lambda phosphatase in the presence of 50 mM EDTA. Assays were incubated at 30°C for 30 min with shaking at 1200 r.p.m. The beads were washed three times in 50 mM Tris pH 7.5, 0.1 mM EGTA and then used in an *in vitro* kinase assay with GST-Parkin Ubl (1–108) as the substrate. Samples were further analyzed as described above.

### 5.11. *In vitro* assay of Parkin E3 ligase activity

Wild-type or Ser^65^Ala Parkin (2 μg) were initially incubated with the indicated amounts of *E. coli*-expressed wild-type or kinase-inactive (D359A) MBP-TcPINK1 in a reaction volume of 25 μl (50 mM Tris-HCl (pH 7.5), 0.1 mM EGTA, 10 mM MgCl_2_, 1% β-mercaptoethanol and 0.1 mM [γ-^32^P] ATP (approx. 500 cpm pmol^−1^); in parallel to confirm the phosphorylation). Kinase assays were incubated at 30°C with shaking at 1050 r.p.m. for 60 min followed by addition of ubiquitylation assay components and Mastermix to a final volume of 50 μl (50 mM Tris-HCl (pH 7.5), 0.05 mM EGTA, 10 mM MgCl_2_, 0.5% 2-mercaptoethanol, 0.12 μM human recombinant E1 purified from Sf21 insect cell line, 1 μM human recombinant UbcH7 purified from *E. coli*, 0.05 mM Flag-Ubiquitin (Boston Biochem) and 2 mM ATP). Ubiquitylation reactions were incubated at 30°C with shaking at 1050 r.p.m. for 60 min and terminated by addition of SDS sample buffer. For all assays, reaction mixtures were resolved by SDS-PAGE. Ubiquitylation reactions were subjected to immunoblotting with anti-FLAG antibody (Sigma, 1:7500), anti-Parkin or anti-MBP antibodies. Incorporation of [γ-32P] ATP into substrates was analysed by autoradiography.

### 5.12. Mapping the site on human Parkin phosphorylated by TcPINK1

GST-Parkin (1 μg) purified from *E. coli* was incubated with 2 μg of either wild-type MBP-TcPINK1 (1–570) or KI MBP-TcPINK1 (D359A) for 60 min at 30°C in 50 mM Tris-HCl (pH 7.5), 0.1 mM EGTA, 10 mM MgCl_2_, 2 mM DTT and 0.1 mM [γ-^32^P] ATP (approx. 20 000 cpm pmol^−1^) in a total reaction volume of 25 μl. The reaction was terminated by addition of lithium dodecyl sulphate (LDS) sample buffer with 10 mM DTT, boiled and alkylated with 50 mM iodoacetamide before samples were subjected to electrophoresis on a Bis–Tris 4–12% polyacrylamide gel, which was then stained with Colloidal Coomassie blue (Invitrogen). Phosphorylated Parkin was digested with trypsin and more than 95 per cent of ^32^P radioactivity incorporated in the gel bands was recovered. Peptides were chromatographed on a reverse phase HPLC Vydac C_18_ column (Cat no. 218TP5215, Separations Group, Hesperia, CA) equilibrated in 0.1% (v/v) trifluoroacetic acid and the column developed with a linear acetonitrile gradient at a flow rate of 0.2 ml min^−1^ and fractions (0.1 ml each) were collected and analysed for ^32^P radioactivity by Cerenkov counting. Isolated phosphopeptides were analysed by LC-MS-MS on a proxeon Easy-nLC nano liquid chromatography system coupled to a Thermo LTQ-orbitrap mass spectrometer. The resultant data files were searched using Mascot (www.matrixscience.com) run on an in-house system against a database containing the Parkin sequence, with a 10 ppm mass accuracy for precursor ions, a 0.8 Da tolerance for fragment ions, and allowing for Phospho (ST), Phospho (Y), Oxidation (M) and Dioxidation (M) as variable modifications. Individual MS/MS spectra were inspected using Xcalibur v. 2.2 software. The site of phosphorylation of these ^32^P-labelled peptides was determined by solid-phase Edman degradation on an Applied Biosystems 494C sequencer of the peptide coupled to Sequelon-AA membrane (Applied Biosystems) as described previously [42].

### 5.13. Large-scale immunoprecipitation of mitochondrial human PINK1 followed by identification of phosphorylated Thr^257^ by mass spectrometry

Ten milligrams of mitochondrial extract from Flp-In T-Rex HEK293 cell lines stably PINK1–FLAG were subjected to immunoprecipitation with anti-FLAG-agarose and then eluted in LDS sample buffer. Samples were boiled with 10 mM DTT, and then alkylated with 50 mM iodoacetamide before being subjected to electrophoresis on a Bis–Tris 4–12% gradient polyacrylamide gel, which was then stained with Colloidal Coomassie blue. Coomassie-stained bands migrating with the expected molecular mass of PINK1–FLAG were excised from the gel and digested with trypsin and samples were analysed either by an Applied Biosystems 4000 Q-TRAP system with precursor ion scanning as described previously [43] or on the LTQ-Orbitrap Velos system with multistage activation.

### 5.14. Large-scale immunoprecipitation of human Parkin followed by identification of phosphorylated Ser^65^ by mass spectrometry

Flp-In T-Rex HEK293 cell lines stably expressing empty vector, wild-type or kinase-inactive PINK1–FLAG were sequentially co-transfected with HA-Parkin, induced with 0.1 μg ml^−1^ of Doxycycline and then incubated with 10 μM CCCP or DMSO control for 3 h before whole-cell lysis. Approximately 30 mg of lysate was subjected to immunoprecipitation with anti-FLAG-agarose and then eluted in LDS sample buffer. Samples were boiled with 10 mM DTT, and then alkylated with 50 mM iodoacetamide before being subjected to electrophoresis on a Bis–Tris 10 per cent polyacrylamide gel, which was then stained with Colloidal Coomassie blue. Coomassie-stained bands migrating with the expected molecular mass of Parkin were excised from the gel and digested with trypsin and samples underwent phosphosite analysis with LTQ-Orbitrap Velos. Individual MS/MS spectra of phosphopeptides were inspected using Xcalibur v. 2.2 software.

### 5.15. N-terminal Edman sequencing

HEK293 cells were transiently transfected with wild-type PINK1-FLAG and then underwent whole-cell lysis. One hundred milligrams of lysate was subjected to immunoprecipitation with anti-FLAG agarose and then eluted in LDS sample buffer. Samples were boiled with 10 mM DTT, and then alkylated with 50 mM iodoacetamide before being subjected to electrophoresis on a Bis–Tris 10 per cent polyacrylamide gel, which was then transferred to Immobilon PVDF (polyvinylidene difluoride) membrane and stained briefly with Coomassie Blue. The band corresponding to the processed form of PINK1 was excised and subjected to Edman degradation in an Applied Biosystems ProCise 494 Sequencer. The resulting HPLC profiles were analysed with Model 610 software (Applied Biosystems).

## 6. Acknowledgements

We thank Eddy Goh, Ron Hay, & Michael Duchen for helpful discussions, Clare Johnson for excellent technical support, the Sequencing Service (College of Life Sciences, University of Dundee, Scotland) for DNA sequencing and the protein production, Ubiquigent for provision of His-UCHL1 and the antibody purification and protein production teams (Division of Signal Transduction Therapy (DSTT), University of Dundee) co-ordinated by Hilary McLauchlan and James Hastie. C.K. is funded by a Parkinson's UK Studentship. A.Z.K. is funded by a J. Macdonald Menzies Charitable Trust Prize Studentship. M.M. is funded by a Wellcome Trust Intermediate Clinical Fellowship. This work was supported by the Medical Research Council (D.R.A.); the Wellcome Trust (M.M.); Parkinson's UK (D.R.A. and M.M.); the Michael J. Fox Foundation for Parkinson's disease research (D.R.A. and M.M.); a Wellcome/MRC PD consortium grant to UCL Institute of Neurology, University of Sheffield and MRC PPU of University of Dundee (D.R.A. and M.M.); and Cancer Research UK (H.W.). We also thank the pharmaceutical companies supporting the Division of Signal Transduction Therapy Unit (AstraZeneca, Boehringer-Ingelheim, GlaxoSmithKline, Merck KgaA, Janssen Pharmaceutica and Pfizer) for financial support.

## Supplementary Material

Supplementary Figures
