# Supplementary material for: PINK1 is activated by mitochondrial membrane potential depolarization and stimulates Parkin E3 ligase activity by phosphorylating Serine 65
Source: Open Biol. 2012 May;2(5):120080. doi: 10.1098/rsob.120080 (PMC3376738; doi:10.1098/rsob.120080)
Supplement: Supplementary Figures [file rsob120080-s1.pdf]

SUPPLEMENTARY FIGURE LEGENDS

Figure S1. Identification of the Ser^{65} phosphorylation site by Edman sequencing and mass spectrometry. Phosphopeptides P2 (A) and P1 (B) from Figure 1C were sequenced by solid-phase Edman degradation using an Applied Biosystems 494C sequencer after the peptides were coupled to Sequelon-arylamine membrane (Applied Biosystems) as described previously (Campbell and Morrice 2002). The amino acid sequence deduced from the LC-MS-MS analysis is shown using the single-letter code for amino acids.

Figure S2. Mapping of PINK1 cleavage site by N-terminal Edman sequencing. HEK293 cells were transiently transfected with wild-type PINK1-FLAG and 100 mg of whole cell lysate immunoprecipitated with anti-FLAG agarose. After electrophoresis, samples were transferred to Immobilon PVDF membrane and stained with Coomassie Blue. (A) Coomassie stained PVDF membrane showing band corresponding to the cleaved form of PINK1 that was excised and subjected to Edman degradation and analysis. The amino acid sequence obtained in the gel band started with FGLGLG (residues 104 – 109). Representative of 3 independent experiments. (B) Sequence alignment of residues around Phe^{104} in human PINK1 showing high degree of conservation amongst higher organisms. Cleavage site indicated by an arrow.

Figure S3. Mass spectrometry confirmation that phosphorylation of PINK1 Thr^{257} is an autophosphorylation site.

Flp-In T-Rex HEK 293 cell line stably expressing wild-type or kinase-inactive PINK1-FLAG (D384A) were treated 10 μM of CCCP for 3 h. (A) Recombinant PINK1 was immunoprecipitated from 10mg of mitochondrial extract for each condition using anti-FLAG-agarose, subjected to 4-12% gradient SDS-PAGE, and stained with colloidal Coomassie blue. (B) The Coomassie-stained bands migrating with the expected molecular mass of PINK1-FLAG were excised from the gel, digested with trypsin, and subjected to LC-MS-MS on an LTQ-Orbitrap mass spectrometer. The Thr^{257} phosphopeptide was only detected in the wild-type PINK1-FLAG band.

Figure S4. Structure of human Parkin Ubl domain. Three views representing a 90° rotation about the y-axis, depict the Ubl domain of Parkin with Ser^{65} highlighted and the contacts it make within 4 Angstroms (PDB code 1IYF).

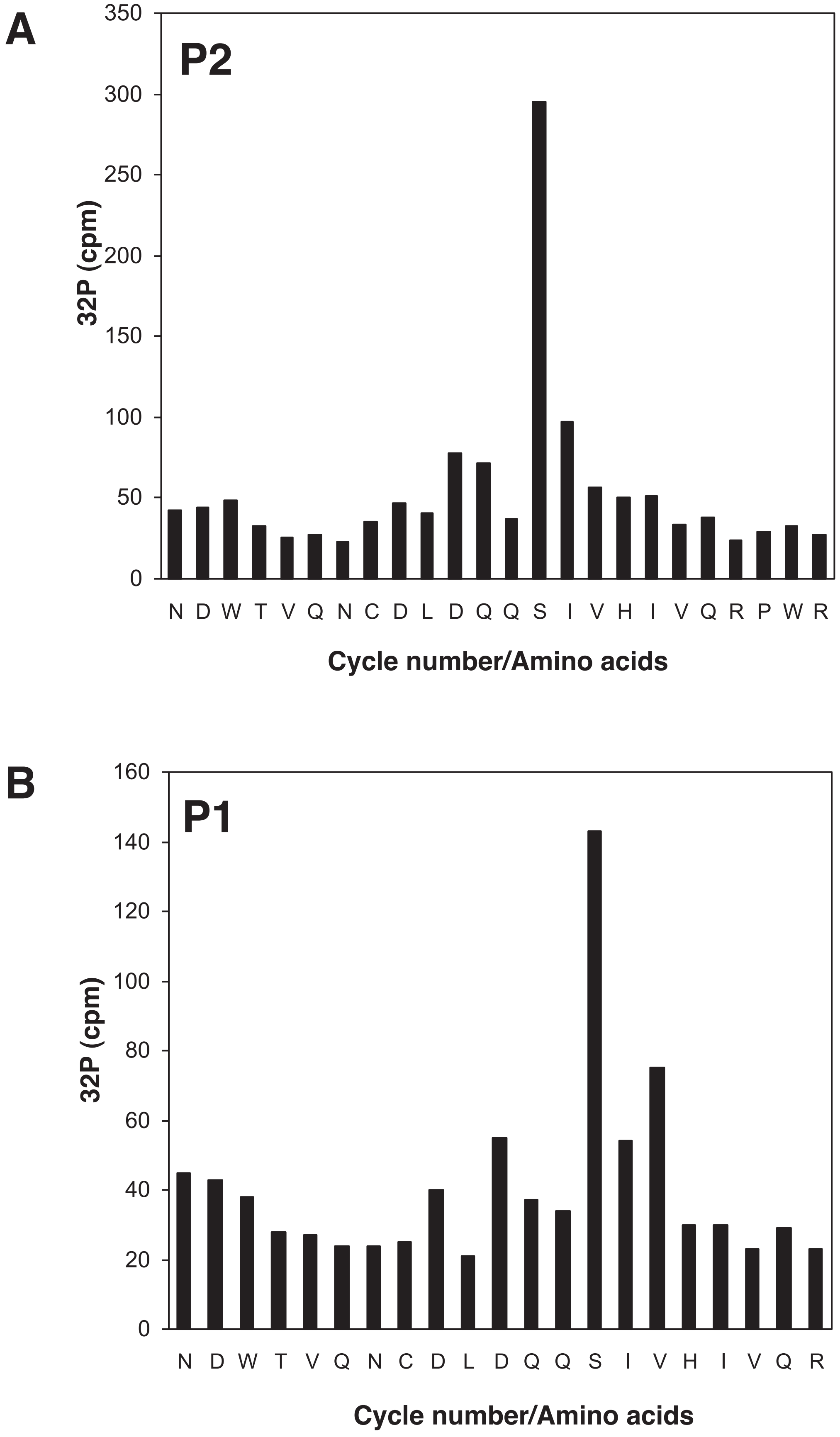
A
P2
32P (cpm)
350
300
250
200
150
100
50
0
N D W T V Q N C D L D Q Q S I V H I V Q R P W R
Cycle number/Amino acids
B
P1
32P (cpm)
160
140
120
100
80
60
40
20
0
N D W T V Q N C D L D Q Q S I V H I V Q R
Cycle number/Amino acids

Figure S1

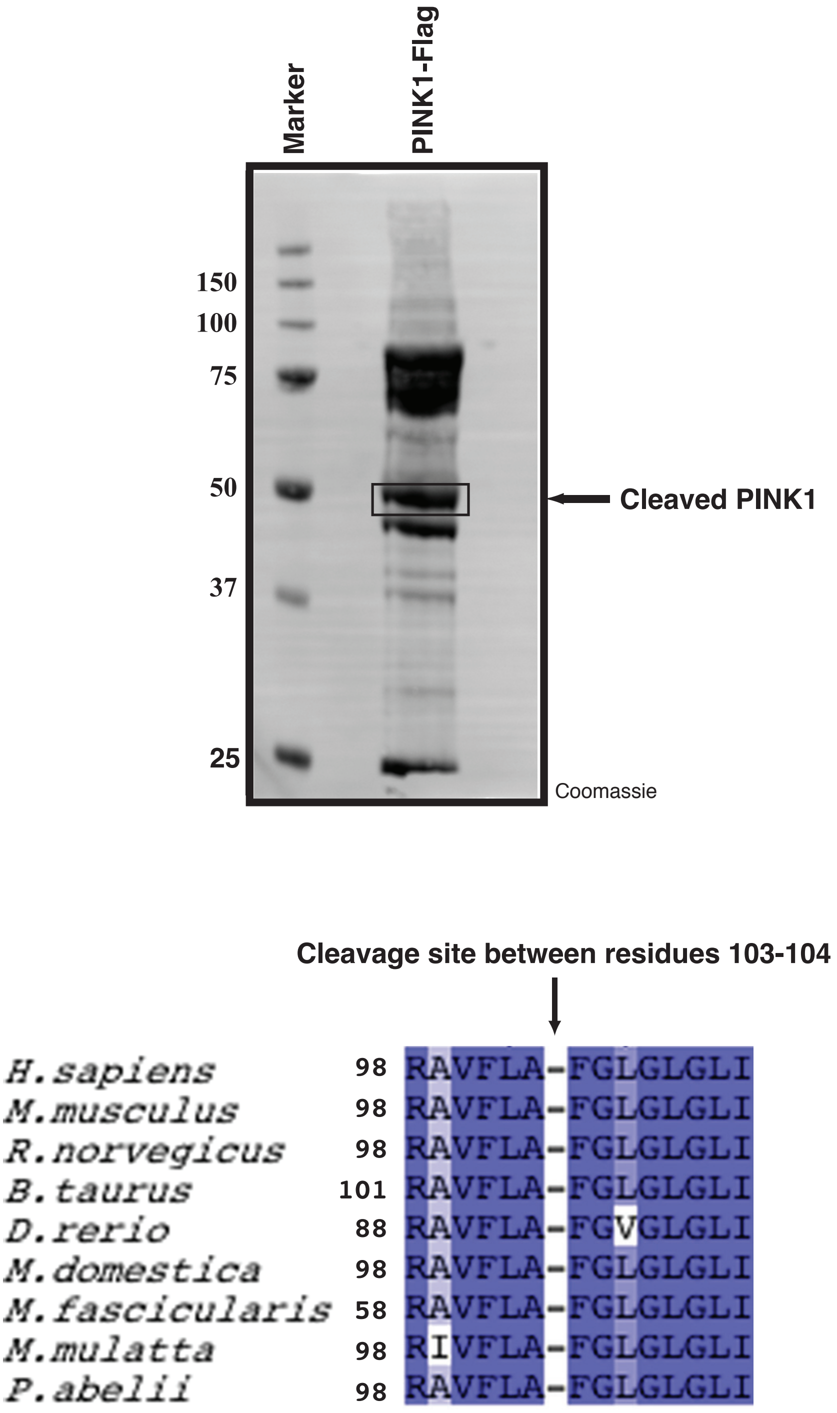

Marker
PINK1-Flag
150
100
75
50
37
25
Cleaved PINK1
Coomassie
Cleavage site between residues 103-104
H.sapiens 98 RAVFLA-FGLGLGLI
M.musculus 98 RAVFLA-FGLGLGLI
R.norvegicus 98 RAVFLA-FGLGLGLI
B.taurus 101 RAVFLA-FGLGLGLI
D.rerio 88 RAVFLA-FGVGLGLI
M.domestica 98 RAVFLA-FGLGLGLI
M.fascicularis 58 RAVFLA-FGLGLGLI
M.mulatta 98 RIVFLA-FGLGLGLI
P.abelii 98 RAVFLA-FGLGLGLI

Figure S2

A

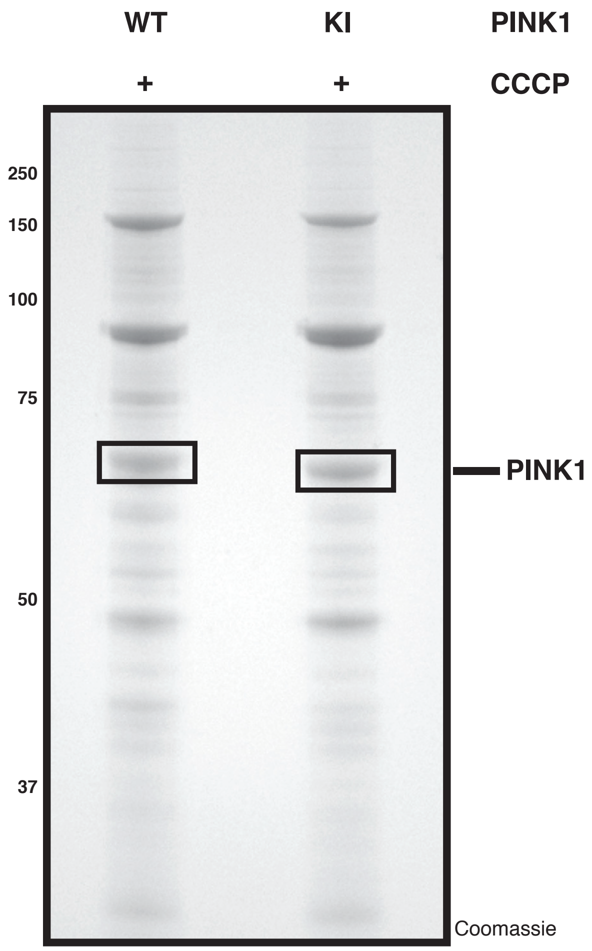

B

Observed Mass (m/z)	Theoretical Mass (m)	Sequence	Phosphorylated Residue	No. of experiments detected
789.38	1576.76	VALAGEYGAV**pT**YRK	257	2

Figure S3

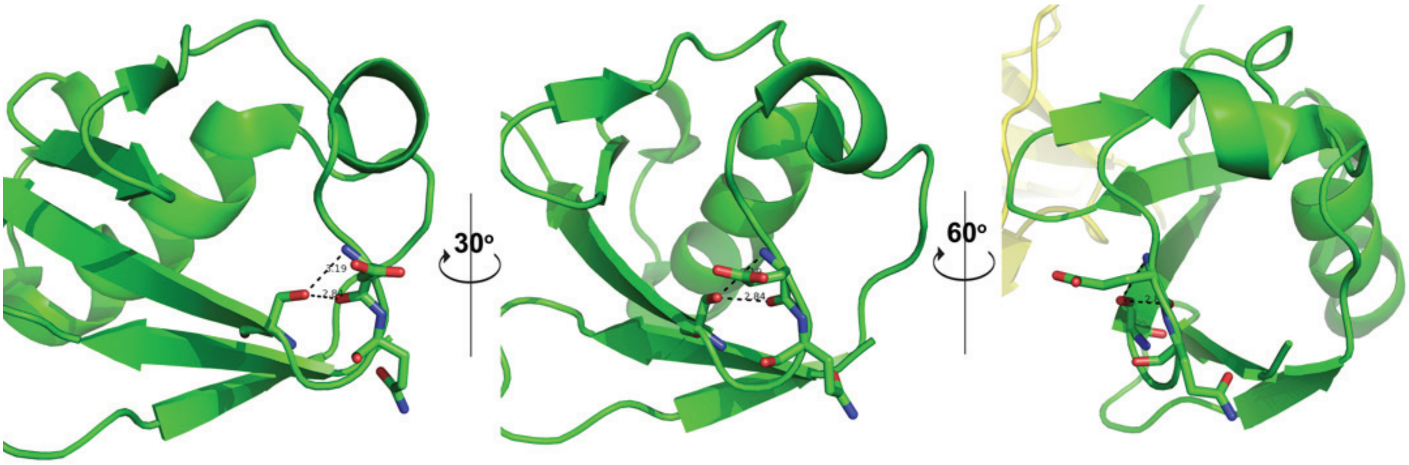

Figure S4
